# Alanates, a Comprehensive Review

**DOI:** 10.3390/ma12172724

**Published:** 2019-08-25

**Authors:** Karina Suárez-Alcántara, Juan Rogelio Tena-Garcia, Ricardo Guerrero-Ortiz

**Affiliations:** Morelia Unit of Materials Institute Research, National Autonomus University of Mexico, 58190 Mexico City, Mexico

**Keywords:** alanates, metal aluminum hydrides, mechanical-milling, hydrogen storage

## Abstract

Hydrogen storage is widely recognized as one of the biggest not solved problem within hydrogen technologies. The slow development of the materials and systems for hydrogen storage has resulted in a slow spread of hydrogen applications. There are many families of materials that can store hydrogen; among them, the alanate family can be of interest. Basic research papers and reviews have been focused on alanates of group 1 and 2. However, there are many alanates of transition metals, main group, and lanthanides that deserve attention in a review. This work is a comprehensive compilation of all known alanates. The approaches towards tuning the kinetics and thermodynamics of alanates are also covered in this review. These approaches are the formation of reactive composites, double cation alanates, or anion substitution. The crystallographic and X-ray diffraction characteristics of each alanate are presented along with this review. In the final sections, a discussion of the infrared, Raman, and thermodynamics was included.

## 1. Introduction

**Figure 1:**
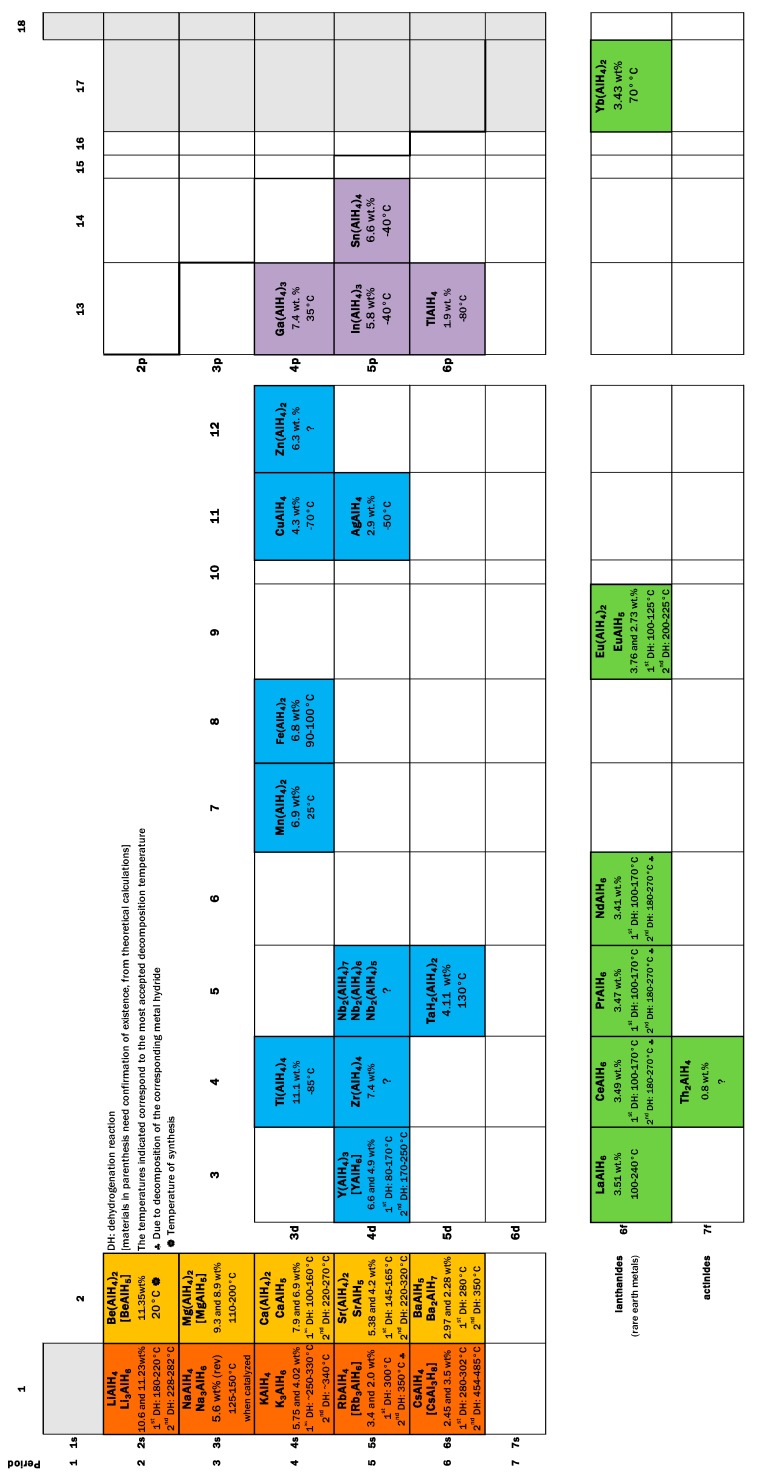
Periodic table of alanates. The reported alanates were collected in this “periodic table”.

Hydrogen storage in solid materials is a relatively new branch of hydrogen technologies. It started during the ’60s of the last century with the systematic study of TiFe alloys and Mg [1,2,3]. The studies on hydrogen storage flourished with the spread of the use of mechanical milling to produce materials or precursors that exhibited improved properties regarding kinetics or thermodynamics [4,5,6]. Another breakthrough was the discovery that certain Ti-compounds made the hydrogen storage/release reversible in NaAlH_4_ [7,8]. Certainly, there are numerous materials that are potentially useful in hydrogen storage. Among them, the family of alanates stands out because of the high hydrogen content, rich chemistry, and the possibility of reversible storage [9]. Alanates (or aluminohydrides) are robust materials; some of them are so well known that prototypes of storage tanks had been constructed (i.e., NaAlH_4_) [10,11,12]. Others, such as Ti(AlH_4_)_4_ or Zr(AlH_4_)_4_, are barely known in terms of crystal structure or thermodynamics [13,14]. Figure 1 presents a “periodic table” of the known alanates with dehydrogenation temperatures.

Alanates are like other hydrogen storage materials, in the sense that no material fulfills all of the requirements of hydrogen capacity, dehydrogenation temperatures, or reversibility. The DOE (Department of Energy, USA [15]) had proposed along several decades the figures of merit for hydrogen storage materials and systems, specifying the type of applications (portable, light-duty vehicles, etc.). In general, high hydrogen storage capacity (6.5 wt.% [15]) and reversibility would prevail as the two fundamental characteristics of hydrogen storage materials. The exigencies of the DOE are very rigorous, particularly for light-duty vehicles applications [15], and they include (not limited to) the quantity of stored/released hydrogen (mass and volume of a complete system, 6.5 wt.% and 5 vol.%), reversibility, kinetics (optimum time to charge a hydrogen tank, 3–5 min), minimum number of cycles of hydrogen charge/discharge (1500), operational temperature (−40 to 85 °C), operational pressure (delivery pressure 5–12 bar), cost of the system (266 USD/kg H_2_), safety, etc. Other factors to be careful with are the thermodynamics (related to the dehydrogenation temperature, but also to the quantity of heat added/removed to/from the system), the onboard efficiency (90%), etc. Moreover, in the future, factors such as recyclability, sustainability, or alanate production from recycled materials [16,17] must also be included as critical factors. However, niche applications for different applications [18] could be developed while using different hydrogen storage materials, including the alanates. These niche applications must meet the particular characteristics of the hydrogen production type and the needs of the final user [18,19]. Nonetheless, the alanate family would allow for the development of new materials. The present work covers the general synthesis procedures, structure, thermodynamics, and hydrogen storage capacity of the known alanates (whenever available). Additionally, double cation alanates or anion substituted materials are also presented and discussed. In the last part of the work, we present a compilation of IR (Infrared) spectroscopy, Raman spectroscopy, and thermodynamics data, along with some general tendencies.

## 2. General Syntheses Procedures

In this section, the synthesis routes are enumerated, describing them in a general way. Further along in this review, more details are presented for each particular alanate. However, all of the alanates have the need for protective atmospheres during handling, synthesis, and actual hydrogenation or dehydrogenation reactions in common. All of the the alanates can be classified as dangerous materials due to their flammability when exposed to oxygen or humidity. Definitely, they ignite and release hydrogen in contact with water, some more violently than others. Thus, great precautions and security measures must be taken when working with alanates.

### 2.1. Syntheses in Organic Solvents

#### 2.1.1. Direct Synthesis

Alanates are frequently synthesized by the reaction of metals or metallic hydrides (e.g., NaH) with Al, H_2_, and a catalyst in organic solvents, such as toluene, hexane, n-octane, ether, diglyme, ether, or tetrahydrofuran (THF) (Equations (1)–(4)) [20,21]. Frequently, a Ti-compound is used as a catalyst. Typically, an excess of Al is used. This method needs the use of moderate to high hydrogen pressure (100–150 bar) and moderate temperatures (120–150 °C); except for LiAlH_4_, which requires a higher pressure (350 bar) [21]. This method can be considered to be highly dangerous due to the explosive mixture of organic solvents, metal hydride, and Al with oxygen and humidity. The materials thus produced require further steps of purification and drying. Frequently, the alanates are kept and sold in THF solution.

M + ½ H_2_ → MH, (1)

MH + Al +3/2 H_2_ ↔ MAlH_4_, M = Li (low conversion), Na, K, Cs (2)

Frequently, Equation (2) is expanded as a two-step reaction with M_3_AlH_6_ as an intermediary [22]:

MH + 1/3 Al + 1/2 H_2_ ↔ 1/3 M_3_AlH_6_, and (3)

1/3 M_3_AlH_6_ + 2/3 Al + H_2_ ↔ MAlH_4_ (4)

#### 2.1.2. Reaction of Metal Hydrides and Aluminum Salts

Another example of lithium alanate synthesis is the reaction of LiH with AlCl_3_ in refluxing ether under an atmosphere of dry nitrogen [23]:

4LiH + AlCl_3_ → LiAlH_4_ + 3LiCl. (5)

This type of reaction is known as “the Schlesinger method”. Despite the simplicity of this reaction, it requires the use of milled LiH (finer than 100 mesh). Additionally, this reaction requires an excess of LiH. Substitution of AlCl_3_ by AlBr_3_ can also be effective [24]. The same reaction outline of Equation (5) can be used with NaH or KH, and AlCl_3_, to produce NaAlH_4_ and KAlH_4_, respectively [24]. However, these reactions need the use of Al(C_2_H_5_)_3_ as a catalyst for the reaction with NaH, and C_6_H_6_-(C_2_H_5_)_2_O as the solvent; and Al(C_2_H_5_)_3_ or (i-C_4_H_9_)_2_AlH as a catalyst for the reaction with KH [24].

The same type of reaction can be applied to M^+2^ alanates, such as Mg(AlH_4_)_2_ (Equation (6)) [25,26,27] or Ca(AlH_4_)_2_ [28], for example:

4MgH_2_ + 2AlCl_3_ → Mg(AlH_4_)_2_ + 3MgCl_2_. (6)

No catalyst is used in the last example.

Some materials are obtained rather as THF adducts when this solvent is used [29]. Frequently, the THF adducts cannot be purified (elimination of THF) without the decomposition of the alanate. The use of protective atmospheres during synthesis can improve the yield of the reactions [29]. A general reaction could be described as:

*n*MH*_x_* + *x*AlCl_3_ → M(AlH_4_)*_x_*+ (*n* − 1)MCl*_x_* (7)

Some of the references for this kind of synthesis are rather old. Initially, this synthesis procedure was not considered for hydrogen storage purposes.

#### 2.1.3. Metathesis of Alanates

Several alanates having one cation or bi-cation have been produced by the metathesis reaction between NaAlH_4_ or LiAlH_4_ and metal halides in organic solvents, such as THF or Et_2_O [30]. One practical reason for this is that NaAlH_4_ and LiAlH_4_ are the only commercially available alanates. This type of reaction dates back from 1950, from the work of Wiberg and Bauer [27], and the reaction can be summarized as:

*n*M1AlH_4_ + M2X_n_ → M2(AlH_4_)_n_ + *n*M1X_n_, (8)

where M1 = Na or Li, M2 = Mg, Ca, or other metals, and X = Cl, Br, I [27,30,31].

Reactions of this type normally are conducted under refluxing conditions from cryogenic to room temperature for several hours or even days. The products usually are adducts of the solvent used, and subsequent operations of purification and drying are required.

### 2.2. Syntheses Assisted by Mechanical Milling

**Figure 2:**
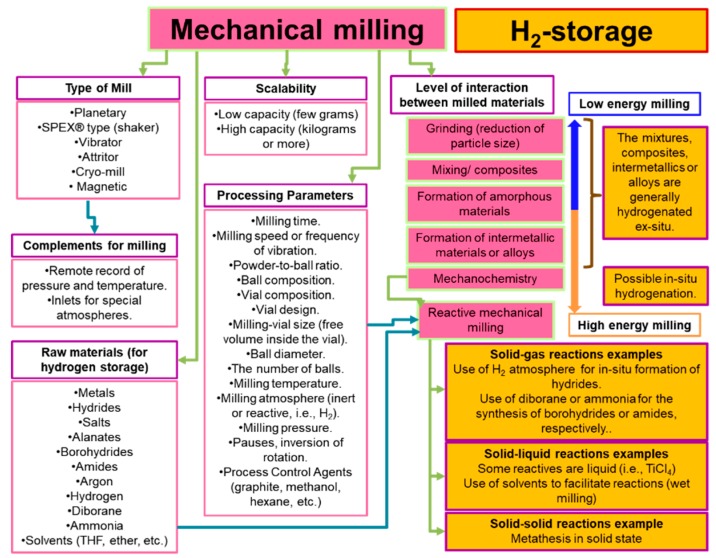
Mechanical milling main concepts.

During the 80s of the last century, the mechanical milling sped up the development of hydrogen storage. We refer both to the study of materials (number of new materials), and the materials themselves towards the storage/release of hydrogen (kinetics of reactions) [5,6]. There are many parameters of mechanical milling. Figure 2 summarizes some of the most important ideas around the mechanical milling that are relevant for the hydrogen storage.

By means of mechanical milling, the same reactions that are described in Section 2.1 can be performed. In most of the cases, the mechanically assisted reactions are faster than the same reactions in solvents. Additionally, the need for solvents is reduced or eliminated. However, some possible problems such as the elimination of side-products (purification) or the contamination by abrasion of balls and vials must be considered. The abrasion of the balls and vial can affect the performance of alanates. Although, an experienced “miller” will know that and would take actions to reduce contamination. These actions can be: (i) Not over-milling. Extended times of milling sometimes can be prejudicial by destroying the alanate, increasing the possibility of abrasion and is a waste of energy. (ii) Check the status of the balls and vial before every milling. (iii) Replace the balls and seals periodically. (iv) Keep the milling vial in good condition. (v) Use compatible materials; there are balls and vials of other materials beyond iron-alloys.

#### 2.2.1. Direct Synthesis by Mechanical Milling

The mechanical milling of the corresponding metal hydride and aluminum and further ex-situ hydrogenation can produce some alanates. Or in-situ by means of a hydrogen atmosphere during the milling. In the ex-situ approach, the hydrogenation of the milled precursors is performed in a specialized reactor (Sieverts type apparatus) to complete Equations (1) and (2). The typical example is the NaAlH_4_ synthesis by means of mechanical milling of NaH and Al, or Na and Al, and further hydrogenation facilitated by additives, dopants, or catalysts. In the in-situ approach (or reactive mechanical milling), the production of alanates can be attained by the solid-gas reaction between the metal hydride, aluminum, additives, and hydrogen. Again, the typical example is the one-step synthesis of NaAlH_4_ [32]. The direct synthesis assisted by mechanical milling is an improvement towards “green chemistry”, including solvent free-synthesis [33,34]. Despite the relative simplicity of this method, it is usually performed only in lab-scale for studies of hydrogen storage.

A third approach is the use of solvents (i.e.; wet ball milling) to obtain a precursor mixture of the alanate [35] or the alanate of interest if a hydrogen atmosphere is used. This methodology requires a drying step.

#### 2.2.2. Reaction of Metal Hydrides and Aluminum Salts under Mechanical Milling

Few examples of Equation (7) by mechanical milling have been reported. This absence of data can be related to the instability of some alanates and the consequent difficulty of their synthesis. Among the examples is the work of Hlova et al. [36,37,38]. They produced the reaction of LiH with AlCl_3_ or NaH with AlCl_3_ in several molar proportions, with the objective of forming AlH_3_. Besides forming AlH_3_, they formed mixtures of LiAlH_4_-LiAlCl_4_-Li_3_AlH_6_ or NaAlH_4_-NaAlCl_4_-Na_3_AlH_6_, respectively, under 345–350 bar of hydrogen pressure. In another example, Dymova et al. reacted 2MgH_2_-AlCl_3_ to form Mg(AlH_4_)_2_ and MgCl_2_ [39].

However, it must be considered that the mechanochemical version of Equation (5), and Equation (7), in general, or any similar reaction that involves hydrides or alanates plus aluminum salts, would compete with the formation of Al, for example [36]:

3MH + AlCl_3_ → Al + 3MCl + 1.5H_2_, (M = Li, Na). (9)

Milling under cryogenic conditions, i.e., with liquid-nitrogen cooling, could be effective in reducing Al formation.

#### 2.2.3. Reaction of Metal Hydrides and Alane under Mechanical Milling 

Another disadvantage of the reaction of metal hydrides and aluminum salts is the loss in hydrogen capacity. This is the result of the formation of salts, such as LiCl, NaCl, or MgCl_2_, which usually are not separated from the products. Alternatively, to avoid the formation of these salts, a reaction of a metal hydride with AlH_3_ has been proposed [40,41,42], for example:

MgH_2_ + 2AlH_3_ → Mg(AlH_4_)_2_ (10)

In some cases, the reaction did not go to completion, and the formation of intermediaries, such as CaAlH_5_, was reported [41]. However, the general, complete, reaction would be:

MH*_x_* + *x*AlH_3_ → M(AlH_4_)*_x_* (11)

The main drawback of this synthesis method is that alane is not a commercial reagent, as such it must be produced in a preliminary step. 

#### 2.2.4. Metathesis of Alanates under Mechanical Milling 

Several alanates have been produced by metathesis promoted by mechanical milling, Equation (8). Once the milling parameters are well established, this method can be very simple and has many advantages. The main advantages include (i) total elimination of solvents and (ii) significant reduction of the reaction time [43]. However, the main disadvantage is that the produced alanate is impure; the product of milling is a mixture of the alanate and salt. The final result is a drastic reduction of the hydrogen capacity. Examples are the production of Mg(AlH_4_)_2_-2NaCl from 2NaAlH_4_-MgCl_2_ [43], Ca(AlH_4_)_2_-2LiCl from 2LiAlH_4_-CaCl_2_ [40], or Eu(AlH_4_)_2_-2NaCl from EuCl_2_-2NaAlH_4_ [44].

## 3. The “Single Metal” Alanates

In this section, experimental and theoretical “single metal” alanates are described. They are ordered in groups according to the periodic table. Alane was also included. At the end of this section, the binary (double cations) alanates are presented. Some of them are well-known, while others are barely developed. This section presents the essential characteristics of synthesis, dehydrogenation reactions, and temperatures, crystallographic data, crystal structures, expected X-ray diffraction patterns, and, in some cases, phase diagrams. 

### 3.1. AlH_3_

The aluminum hydride or alane is a material with high hydrogen content (10 wt.%). A 10 wt.% of hydrogen is very attractive; it meets the DOE targets of 6.5 wt.% of hydrogen for mobile applications. Even more, the low dehydrogenation temperature makes the alane, in principle, compatible with polymer exchange membrane fuel cells (PEMFC) applications. AlH_3_ is typically produced by the reaction of LiAlH_4_ with AlCl_3_ in an organic solvent, such as THF or Et_2_O [45]:

3LiAlH_4_ + AlCl_3_ + *n*Et_2_O → 4AlH_3_·*n*Et_2_O + 3LiCl↓. (12)

Instead of LiAlH_4_, LiH was used in the early studies of this reaction [45]. The product is an adduct that must be separated from the solvent. An excess of LiAlH_4_ or some LiBH_4_ is added to the reaction mixture to improve the time and temperature of desolvation [45,46]. The solvent-free mechanosynthesis of AlD_3_ was performed while using cryomilling 3LiAlD_4_ + AlCl_3_ at a low temperature (−196 °C). This conditions eliminated the competing reaction towards the formation of Al and LiCl [47,48]. This synthesis allowed for the determination of the structures of α-AlD_3_ and α’-AlD_3_. Mechanical milling of 3LiAlH_4_ + AlCl_3_ at room temperature also can produce the alane by using high pressures of hydrogen (210 bar) or inert gas (125 bar of He or 90 bar of Ar) [49]. Alternatively, the alane can be produced by the electrochemical reaction of LiAlH_4_ or NaAlH_4_ with or without LiCl as an electrocatalytic additive and with or without hydrogen atmosphere. The general reactions involved are [50,51,52]:

3AlH_4_^−^ +Al + *n*THF → 4AlH_3_·*n*THF + 3e^−^, anode of Al (13)

3(M^+^ PdH + e^−^→ MH + Pd), cathode of Pd, PdH_2_ (14)

According to reports, the alane has seven polymorphs, and here we present the four most frequently reported (Table 1) [46]. The energy of phase transition between these polymorphs is low: around −1 to −2 kJ/mol H_2_; thus, the phase transitions occur spontaneously at room temperature (adding complications to the crystal structure determination) [53]. The common structure of the alanes is corner-shared (AlH_6_) octahedra [54]. 

The reported formation enthalpy of alane is around −6 to −9 kJ/mol H_2_; thus, an equilibrium pressure of the order of 10^5^ bar at room temperature is expected [53]. However, the minimum hydrogen pressure, experimentally observed and calculated, which is necessary for the formation of the alane from the elements is about 7000 bar at room temperature [55]. Thus, on-board regeneration of alane for hydrogen storage in automotive applications is definitely out of the picture. Recently, a report on nanoconfined AlH_3_ indicates partial re-hydrogenation at 150 °C and 60 bar [56]. Nanoconfinement reduces the hydrogen content; however, it must be explored as a way to reach reversibility.

**Figure 3:**
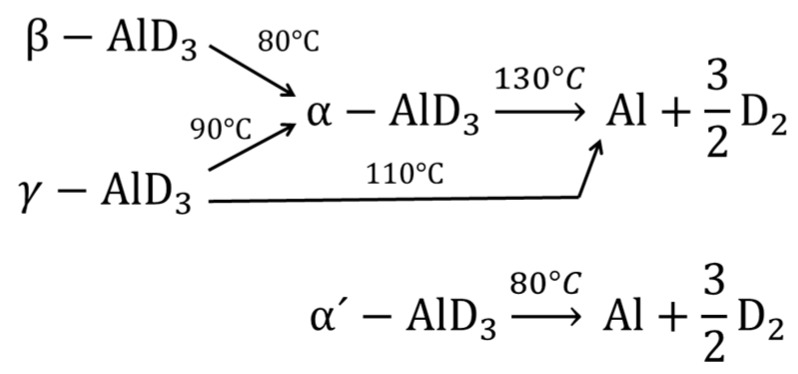
Dehydrogenation pathway of several deuterated alanes (adapted from [62]).

Dehydrogenation enthalpies range from −5 to 6 kJ/mol H_2_ for the different polymorphs [57], thus near room temperature decomposition would be expected. Dehydrogenation temperatures are observed in the range of 150–200 °C [53,58]; however, ball milling has reduced the dehydrogenation temperature below 100 °C [59]. Alane is considered as a metastable hydride, due to the formation of surface oxides, which protect against to further oxidation or decomposition. The surface oxides impose a kinetic barrier to decomposition [58,60]. In particular, for the alane, the passivation is somehow beneficial, reducing decomposition during its storage and handle in the laboratory. However, in general, passivating surface oxidation is a problem. It is challenging to reduce the oxygen and humidity content of protective atmospheres (argon) until acceptable values (<10 ppm) for hydrogen storage applications. This means that a hydrogen storage system that is based on alanates (and hydrides in general) must have proper filtering, trapping, or regenerative systems to reduce oxygen and humidity content, which can be costly. Ball milling of alane exposes new, fresh, and non-oxidized surfaces that improve the kinetics of the dehydrogenation reaction [61]. The dehydrogenation pathways, as proposed by Sartory et al., are presented in Figure 3 [62]. 

The thermal dehydrogenation of alane was improved by the use of simple hydrides, such as LiH [63]; otherwise, AlH_3_ is useful in reducing dehydrogenation temperature or improving dehydrogenation kinetics when added to MgH_2_ or LiBH_4_ [64,65].

**Figure 4:**
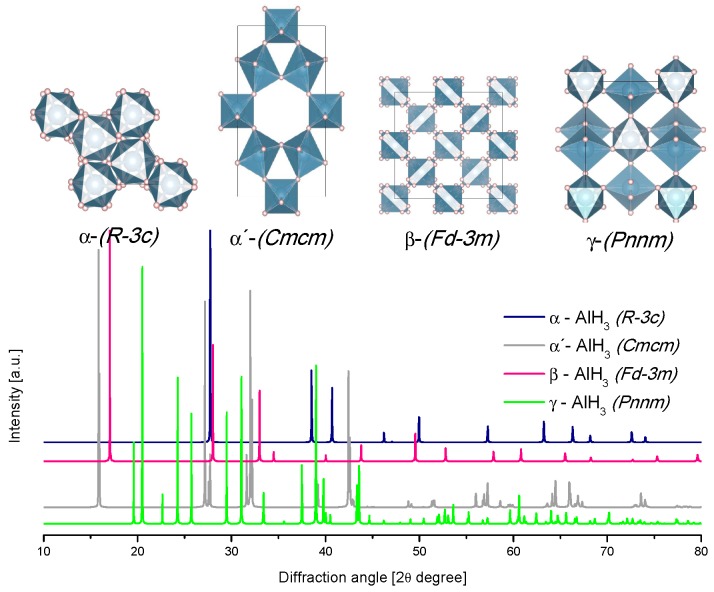
Crystal structure of several alanes and their calculated diffraction patterns (λ = Cu_kα1_).

In the present review, the crystal structures and the calculated X-ray diffraction powder patterns (powder cell 2.3 and mercury software 3.8) are presented for visual comparison. The first of them correspond to the alane polymorphs (Figure 4).

### 3.2. Alanates of Group 1

#### 3.2.1. Lithium Alanate

The LiAlH_4_ has the highest hydrogen content of all alanates, 10.6 wt.%; this is due to the lightness of Li atoms. LiAlH_4_ and NaAlH_4_ are the only commercially available alanates; their cost, of course, is not low enough for massive applications. Both of them are currently produced while using direct synthesis in an organic solvent. Mechanochemical production of LiAlH_4_ by the milling of LiH and Al under hydrogen atmosphere has given minimal results [69]. 

Pure and not milled LiAlH_4_ undergoes a melting transition, at 160–180 °C before undergoing a first dehydrogenation reaction to give Li_3_AlH_6_ and Al at 180–220 °C, Equation (15). A second dehydrogenation reaction is observed to occur at 228–282 °C to give LiH and Al, Equation (16) [70,71]:

LiAlH_4_ → 1/3 Li_3_AlH_6_ + 2/3 Al + H_2_ (15)

1/3 Li_3_AlH_6_ → LiH + 1/3Al + ½ H_2_ (16)

Global reaction: LiAlH_4_ → LiH + Al + 3/2 H_2_ (17)

Together, both reactions provide for a hydrogen release of 7.9 wt.%. The third dehydrogenation step, i.e., the LiH decomposition is beyond any practical hydrogen storage operational temperature. Ball milling and the use of additives have reduced the dehydrogenation temperature of LiAlH_4_ [72]. The list is extensive among the additives. However, the use of Ti-salts, TiCl_3_·1/3AlCl_3_, [73], or NbF_5_ [74] stands out. Data on apparent activation energies indicate an effective reduction of this parameter upon the use of additives [74]. Blanchard et al. proposed a reduction or elimination of an induction period (slow production rate of Al or Li_3_AlD_6_ nuclei) during the decomposition of LiAlD_4_ as the action mode of the additives [75].

**Figure 5:**
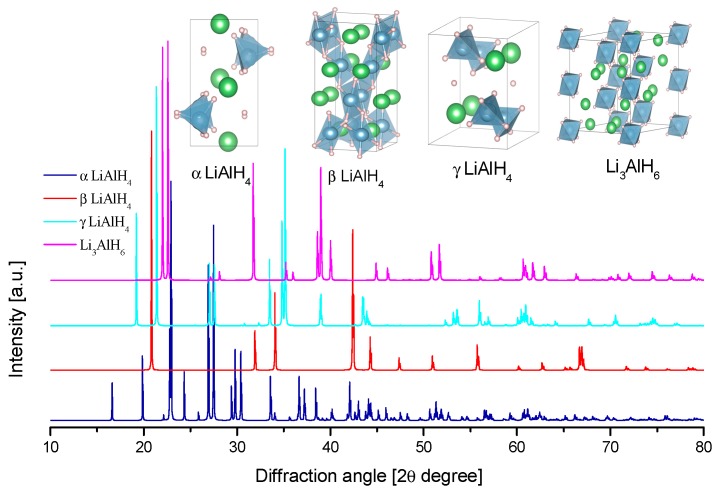
Crystal structure of lithium alanates and their calculated diffraction patterns (λ = Cu_kα1_).

A common characteristic of all alanates is the covalent character of the Al–H bond, while the interaction between [AlH_4_]^−^ or [AlH_6_]^3−^ and M^n+^ is ionic [76]. The crystal structure of α-LiAlH_4_ (α-LiAlD_4_) and Li_3_AlH_6_ (Li_3_AlD_6_) is well-known, as determined both experimentally and by first-principles (Table 2 and Figure 5) [77,78,79]. Additionally, two high-pressure phases, β-LiAlH_4_, and γ-LiAlH_4_, have been described [76,80]. The α-LiAlH_4_ to β-LiAlH_4_ transition is expected to occur between 26,000 [76] −71,500 [69] bar. The β-LiAlH_4_ to γ-LiAlH_4_ transition is expected at 338,000 bar [69]. These pressures are far away from any application in hydrogen storage. 

**Figure 6:**
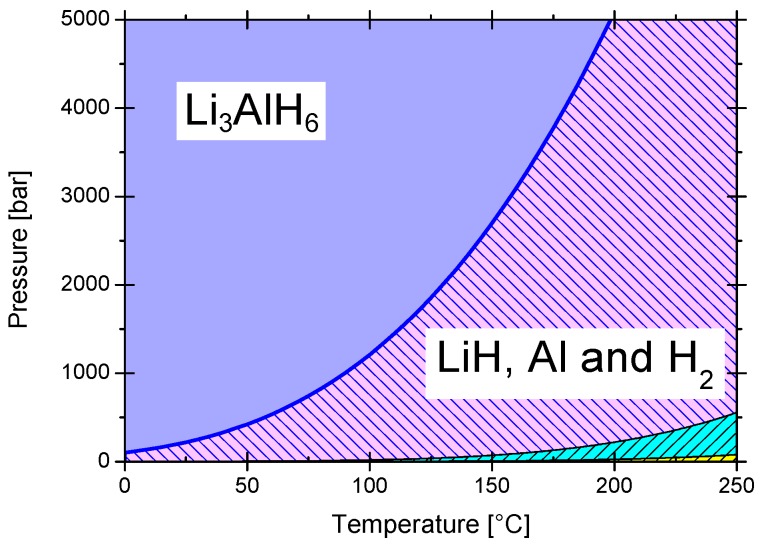
Phase diagram of LiH/Al/H_2_ and Li_3_AlH_6_. The blue line represents the equilibrium. Data adapted from reference [86]: $\ln\left(p\right)=-\frac{0.22}{RT}+13.89$; where (in the original formula) *p* is in atm, *T* in Kelvin and $\Delta H_{R}=0.22\;\mathrm{eV}$. For visual reference (bottom and right) the equilibrium of Ti-doped Na_3_AlH_6_ (blue zone) and NaH + Al (yellow zone) phases were included [88].

LiAlH_4_ is a well-known hydrogen storage material due to its facile dehydrogenation, but practically impossible complete rehydrogenation at moderate conditions. Few examples of successful rehydrogenation were observed by transferring the dehydrogenated products to an organic solvent and then exposing them to a hydrogen atmosphere. Among the examples is the rehydrogenation in Me_2_O at room temperature, 100 bar hydrogen pressure, and 24 h stirring [82,83]. Another reported approach was performing the hydrogenation/dehydrogenation reactions in organic solvent [84]. The experiments and calculations indicate that the LiAlH_4_ rehydrogenation is thermodynamically restricted [85]. The theoretical (*ab-inito*) calculations indicate that the dehydrogenation products of LiAlH_4_ are thermodynamically favored [86]. Ke et al. give the (*T*, *p*) stability diagram of LiH and Al versus Li_3_AlH_6_; these data indicate the need for very high pressures to produce Li_3_AlH_6_ from 3LiH + Al + 3/2H_2_ (Figure 6). In a (*T*, *p*) phase diagram for LiH/Li_3_AlH_6_ and Li_3_AlH_6_/LiAlH_4_, Jang et al. demonstrated an equilibrium pressure of about 10^5^ bar for Li_3_AlH_6_/LiAlH_4_ in a wide range of temperatures [87]. Unfortunately, no equation was given to reproduce that equilibrium line. On the other hand, the equilibrium pressure of the direct and reverse reaction in THF;

(18) $$\mathrm{LiH}+\mathrm{Al}+3/2H_{2}\;\overset{\mathrm{THF}}{↔}\;{\mathrm{LiAlH}}_{4}\cdot 4\mathrm{THF},\;$$

is in the range of 1–2 bar at 80–90 °C [84]. This equilibrium has been studied in a very limited way. Perhaps, a liquid system of hydrogen storage based on LiAlH_4_ deserves more attention. 

#### 3.2.2. Reactive Mixtures (Composites) with LiAlH_4_

Reactive mixtures of hydrides have been proposed as a way to tailor the dehydrogenation temperature or improve rehydrogenation in borohydrides [89]. In this approach, two (or recently more) hydrides (simple or complex) are mixed; and, under suitable dehydrogenation conditions, they react with each other. The dehydrogenation is modified, including the dehydrogenation pathway, temperature, kinetics, and reversibility. Notably, the dehydrogenation temperature of composites is sensitive to the way of mixing of materials and the history of the composite; i.e., time and conditions of mixing, purity of reagents, cycling, etc. In the past decade, the research on LiAlH_4_ has extended, intentionally or not, to the formation of reactive mixtures (composites). Relevant published work is compiled in the next sections.

##### Composites of LiAlH_4_-MgH_2_

Along the last decade, several LiAlH_4_-MgH_2_ composites have been studied for hydrogen storage [90,91,92,93,94,95]. The main results coincide in that the dehydrogenation pathway is marked by three steps, the usual two of LiAlH_4_ and one of MgH_2_. The temperature of these dehydrogenation steps is somewhat reduced compared to the pure components. Even more, the use of additives, such as TiH_2_ [96], TiF_3_ [90], MnFe_2_O_4_ [91], or HfCl_4_ [93], reduced approximately up to 60 °C the dehydrogenation temperatures as compared to the mixture without additives. The role of the additives is to reduce the activation energy of dehydrogenation [93]. Other points of coincidence are the formation of Mg-Al and Li-Mg compounds of relatively varied stoichiometry after dehydrogenation and the occurrence of partial reversibility dominated by MgH_2_ rehydrogenation without indications of LiAlH_4_ rehydrogenation. 

##### Composites of LiAlH_4_-LiBH_4_

LiBH_4_ is as a potential hydrogen storage material due to its high hydrogen content. However, the dehydrogenation/hydrogenation high temperature and pressure prevent its use in a pure form. Thus, LiBH_4_ has been mixed with a variety of chemicals, including LiAlH_4_, for the formation of binary composites [97,98,99,100]. Additionally, ternary composites of the type LiAlH_4_-LiBH_4_-MgF_2_ have been proposed [101]. In this regard, the possibilities of ternary composites are almost infinite. There are a lot of factors to consider, such as the selection of the composites, the relative composition, milling conditions, etc. Systematic studies are missing, noticeably by the difficulty and enormous of the task. The LiAlH_4_ did not survive the milling process in many catalyzed mixtures, resulting in a mixture of LiBH_4_, LiH, and Al [97]. Mao et al. proposed that LiAlH_4_-LiBH_4_ doped with TiF_3_ has a reduced dehydrogenation enthalpy as compared with pure LiBH_4_ [99]. The reported studies coincide in a two-step dehydrogenation pathway and a reduction of the dehydrogenation temperatures, especially if a catalyst, such as TiF_3_, is used [99]. The first reaction is the decomposition of LiAlH_4_ at temperatures around 100 °C. The second step is the decomposition of LiBH_4_. However, the presence of Al directs the formation of AlB_2_ [98]:

2LiBH_4_ +Al ↔ 2LiH + AlB_2_ + 3H_2_ (19)

The rehydrogenation of the LiAlH_4_-LiBH_4_ mixtures was proven to occur at various conditions of pressure and temperature, among them 40, 70, and 85 bar, and 350, 400, and 600 °C [97,98,99]. The rehydrogenation is directed to the formation of LiBH_4_, since no rehydrogenation of LiAlH_4_ has reported. While using NaBH_4_ instead of LiBH_4_ conduces to similar conclusions; a two-step dehydrogenation with reduced temperature as compared with pure materials, the presence of AlB_2_ after dehydrogenation, and partial hydrogenation due to the formation of NaBH_4_ [102].

However, Xia et al. [103] reported the formation of Li_3_AlH_4_ and LiBH_4_ in successive rehydrogenations of 2LiBH_4_-LiAlH_4_ confined in mesoporous carbon scaffolds (up to 8.5 wt.% content, rehydrogenation at 500 °C, 100 bar, 10 h, seven cycles). Confinement in meso or nanoporous materials is another strategy for reducing the dehydrogenation temperature and improving the reversibility. However, a reduction in the hydrogen capacity is expected. Other confinement effects are [104,105,106,107]: (i) The reduction or total elimination of the loss of critical elements, such as B in the borohydrides. (ii) Reduction of the diffusion pathways of several species. (iii) Interaction with the meso or nanomaterials supports (can be of catalytic type). (iv) Large surface areas. (v) Reduction of the activation energies. The confinement as a strategy for improving hydrogen storage properties depends on several factors, such as: (i) the material used for confinement (carbons, nanocarbons, zeolites, graphene, silica, etc.) (ii) The history of the confined material. (iii) The way of infiltration (and drying if necessary). (iv) The size of the porous. (v) Functionalization of the surface of the support material. Confinement is a universe of possibilities, and it deserves a mayor review that is beyond the scope of the present report on alanates. 

##### Composites of LiAlH_4_-LiNH_2_

The LiAlH_4_-LiNH_2_ composites have also been studied [108,109,110,111,112,113]. The first dehydrogenation steps are the decomposition of LiAlH_4_ to Al and LiH. Then its dehydrogenation products react with LiNH_2_. Here, less consensus can be found (compared to the previous examples of LiAlH_4_ composites), and several reactions, mechanisms, and intermediaries have been proposed, for example:

Chen et al. proposed the reaction of LiNH_2_ with Al as [108]:

LiNH_2_ → ½ Li_2_NH + ½ NH_3_ (20)

Al + NH_3_ → AlN + 3/2 H_2_ (21)

Evidently, due to NH_3_ production, this method cannot be intended for proton-exchange membrane (PEM) fuel cells. 

Dolotko et al. [111] indicated that reaction (21) has a minor contribution to the dehydrogenation reaction, instead, they proposed that LiNH_2_ reacts with both LiH and Al:

2LiNH_2_ + LiH + Al → Li_3_AlN_2_ + 5/2 H_2_, (22)

and the overall reaction was proposed as:

LiAlH_4_ + LiNH_2_ → ½ Li_3_AlN_2_ + ½ Al + ½ LiH +11/4 H_2_ (23)

Lu et al. proposed that the overall reaction is [112]:

2LiAlH_4_ + LiNH_2_ → 2Al + Li_2_NH + LiH + 4H_2_ (24)

Jepsen et al. studied LiAlH_4_-LiNH_2_ composites in several molar proportions [113]. The intermediary Li_4−*x*_Al*_x_*(NH)_2−2*x*_N_2_ formed when the LiAlH_4_-LiNH_2_ ratio was 1:1.5, 1:2, and 1:2.5. This study supports that the LiNH_2_ reacts with LiH to form Li_2_NH and H_2_. The main differences among the studies are mainly the molar proportions and milling conditions. This last parameter ranged from some minutes of manual milling in a mortar to several hours of mechanical milling. The use of additives, such as transition metal chlorides reduced, approximately 30 °C, the dehydrogenation temperature [114]. Regarding the reversibility, partial reversibility was proven while using rather hard conditions, i.e., 180 bar and 275 °C [111] or 100 bar and 425 °C [113]. However, the reversibility does not rely on the formation of LiAlH_4_. 

#### 3.2.3. Sodium Alanate

NaAlH_4_ is the most important and studied alanate. NaAlH_4_ is used as a reducing agent in many reactions unrelated to the hydrogen storage. Due to the work of Bogdanović et al. on the use of catalysts or additives, the regeneration of NaAlH_4_ is possible in the solid-state. Thus, this material has been seriously considered for hydrogen storage [7,88,115]. The dehydrogenation and rehydrogenation reactions are [7,116]:

NaAlH_4_ → 1/3 Na_3_AlH_6_ + 2/3 Al + H_2_ (25)

1/3 Na_3_AlH_6_ → NaH + 1/3Al + ½ H_2_ (26)

Global reaction: NaAlH_4_ → NaH + Al + 3/2 H_2_ (27)

Reactions (25) and (26) account for 5.6 wt.% of reversible hydrogen storage. Uncatalyzed NaAlH_4_ experiences a solid to liquid phase transition before dehydrogenation. Meanwhile, in catalyzed NaAlH_4_, the dehydrogenation temperature is generally lower than the melting point [117]. The first dehydrogenation step occurs at 210–220 °C. Meanwhile, the second step occurs at approximately 250 °C [117]. 

**Figure 7:**
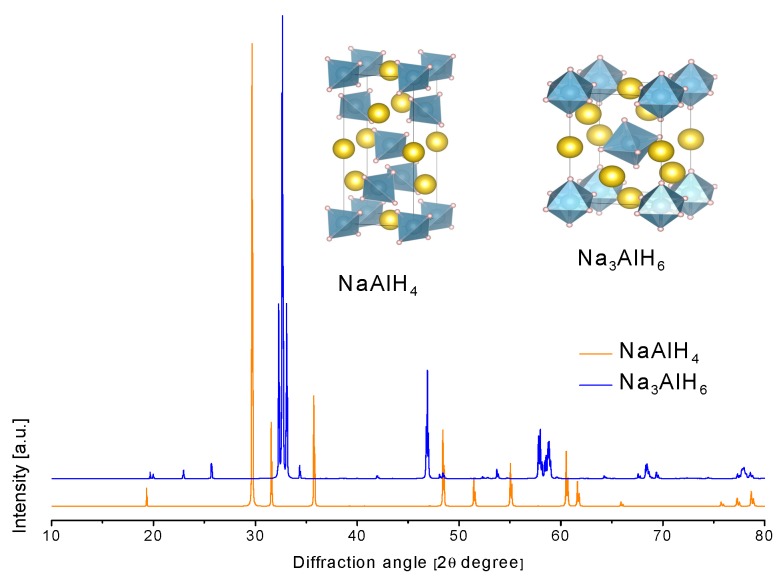
Crystal structure of sodium alanates and their calculated diffraction patterns (λ = Cu_kα1_).

The crystal structure of NaAlH_4_ was determined in 1979 (Table 3 and Figure 7) [118]. The NaAlH_4_ consists of [AlH_4_]^−^ tetrahedra, with the Na atoms that are surrounded by eight [AlH_4_]^−^ tetrahedra in a distorted square antiprism geometry [119,120].

**Figure 8:**
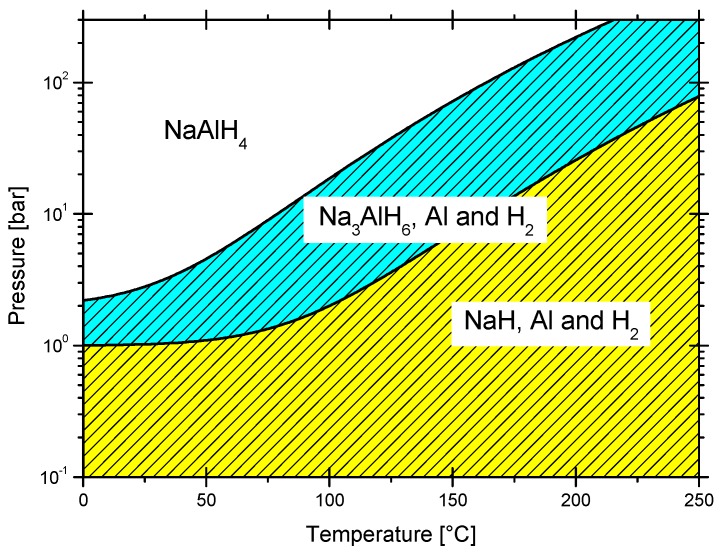
Phase diagram of Ti-doped (Ti(OBu)_4_) NaAlH_4_, Na_3_AlH_6_, and NaH + Al. Na_3_AlH_6_/NaAlH_4_: $\ln\left(\frac{p_{eq}}{p}\right)=-\frac{37\;\mathrm{kJ}\cdot {\mathrm{mol}}^{-1}}{RT}+\frac{122\;J\cdot {\mathrm{mol}}^{-1}\;K^{-1}}{R}$. NaH and Al/Na_3_AlH_4_: $\ln\left(\frac{p_{eq}}{p}\right)=-\frac{47\;\mathrm{kJ}\cdot {\mathrm{mol}}^{-1}}{RT}+\frac{126\;J\cdot {\mathrm{mol}}^{-1}\;K^{-1}}{R}$ [88,133].

The NaAlH_4_ and Na_3_AlH_6_ dehydrogenation enthalpies are well known (37 and 47 kJ/mol H_2_, respectively, Ti-doped) [88]. These values mainly indicate a kinetic restrain for hydrogenation/dehydrogenation reactions, rather than a thermodynamic difficulty (see Section 7 for details on dehydrogenation enthalpies). A phase diagram NaH + Al/Na_3_AlH_4_/NaAlH_4_ can be constructed from these data (Figure 8), [88] which indicates that equilibrium pressures at moderate temperatures are technically achievable, particularly if a catalyst is used. Since the work of Bogdanović, literally, thousands of papers have been published about different catalysts, variations in compositions or variations of the synthesis procedure [123]. NaAlH_4_ can be produced by all of the methods that are described in Section 2 in several conditions of pressure and temperature at laboratory scale by the use of a catalyst [32,35]. Among the catalysts, dopants, or additives, the list includes, but is not limited to: chlorides of the first and second row of transition metals [124], lanthanide-oxides, such as La_2_O_3_, CeO_2_, Sm_2_O_3_, and Gd_2_O_3_ [125], titanium compounds, such as Ti(OBu)_4_ [88], TiCl_3_ [7], TiF_3_, TiCl_4_ [117], TiB_2_ [126,127], TiN [128], TiCl_3_-1/3AlCl_3_ [129], chlorides of Sc and Ce [130], or carbon nanomaterials [131]. The effectiveness of these materials as reaction accelerators is related to the additive level, the addition technique (milling, impregnation with solvent, CVD, etc.), structure, and morphology [127,132]. 

##### Role of Catalyst

Among the extensive list of materials tested as catalysts, dopants, or additives for improving hydriding and dehydriding reactions of NaAlH_4_, the Ti, Sc, and Ce compounds stand out due to their effectiveness [132]. However, most of the theoretical and experimental studies to unravel the action mode of the catalyst have focused on Ti-compounds [134]. Nevertheless, after almost 20 years of the discovery of the benefits of using a catalyst, some fundamental questions are still not adequately addressed. Here are some points to consider:

1. Morphology/particle size effects. Beattie et al. demonstrated that Ti-doped NaAlH_4_ particles presented few morphological changes as compared with un-doped materials [135]. By-products of the addition of materials, such as TiCl_3_, i.e., Ti-Al alloys, and NaCl, can act as grain refiners for Al and NaH phases, keeping particle sizes small [136]. In general, much effort is put to reduce particle sizes and to avoid the sintering of particles, and thus maintaining the hydriding/dehydriding performance.
2. Location of Ti and substitution of atoms. The Ti atoms can be located in the bulk, in interstitial positions, at the subsurface, or the surface. The Ti preferred position depends on the doping level and synthesis technique (impregnation vs. ball milling), or in theoretical calculations, the choice of reference states. The Ti atoms can be located in NaH, Al, Na_3_AlH_6_, or NaAlH_4_ phases. Theoretical studies have been performed basically to include all of these possibilities. Some studies have unraveled the interactions of Ti (or Ti-compounds) with NaH and Al. Other reports indicated interactions of Ti (or Ti-compounds) with Na_3_AlH_6_ and NaAlH_4_. Contradictory results/conclusions frequently come across.
  Additionally, many studies point to atom substitution and formation of defects. The replacement of Al by Ti in NaAlH_4_ could be possible, yet this configuration is metastable [137,138]. Løvvik situates the substitution in the second metal layer from the surface [137,138]. Other DFT calculations suggest that the most frequent Ti-defect in NaAlH_4_ is a defect that is formed by the substitution of Al by Ti and the addition of two hydrogen ions; this defect has a −1 charge [139].
  The substitution of Na by Ti and other metal atoms also has been investigated. Marashdeh et al. classified the catalysts as “good” (Sc, Ti, Zr) and “bad” (Pt, Pd) according to their ability to exchange places with a Na atom on a (001) surface of NaAlH_4_ [140]. In the “zipper model”, Ti-species, at the surface or at a grain-boundary, displace subsurface Na atoms and eject them to the NaAlH_4_ surface. Subsequently, the Na atoms react quickly with other species and destabilize the surface, which returns the Ti-species to a surface location [134,140]. For Na_3_AlH_6_, Michel et al. found a competition between Ti substitution on the Na sites (+1 charge defect) and Ti substitution on the Al site, with an additional bound to H atom (neutral site) [139].
  For the hydrogenation reaction, the reports indicate that Ti near an Al surface (subsurface) promotes H_2_ dissociation and H spillover on the Al surface [141]. Wang et al. remind us, in favor of this role of subsurface Ti, that metallic aluminum does not absorb diatomic hydrogen from the gas phase by itself. Meanwhile, atomic hydrogen strongly reacts with aluminum surfaces to form alanes [142]. Thus, subsurface Ti would promote H_2_ dissociation and enhance H mobility and adsorption [142]. These effects constitute essentially the “hydrogen pump” action mechanism that was proposed for Ti [134]. Theoretical calculations of subsurface Sc, V or Nb substitution of Al indicate that these materials could also perform as a catalyst [143]. Wang et al. also remind us that Ti, Zr, V, Fe, Ni, Nb, Y, La, Ce, Pr, Nd, and Sm are expected to be good catalysts based on their ability to “mix” well with Al [142].
3. Progressive changes of the oxidation state of Ti-species. While Ti^+3^ species is the most recurrent initial oxidation state of the Ti-catalyst, several reports conclude that the oxidation state changes to Ti^0^, followed by the formation of Ti*_x_*-Al*_y_* alloys, and finally the formation of Al_3_Ti [134,144,145,146]. However, Al_3_Ti seems to be an inefficient catalyst, as compared to other Ti or Ti-compounds [134,147]. Perhaps the formation of Ti*_x_*-Al*_y_* alloys and Al_3_Ti is the reason for the long-term (after hydriding/dehydriding cycling) decay of hydrogen storage capacity [148].
4. Formation of Ti-Al-H complexes. Theoretical calculations suggest that the replacement of Na by Ti near o connected with [AlH_4_]^−^ would lead to the formation of Ti-Al-H complexes that can help during the dehydrogenation/rehydrogenation reactions [149,150,151]. TiAl_2_H_7_ and TiAl_2_H_2_ are two optimized structures of the Ti-Al-H complexes [150]. The effect of the Ti-Al-H complexes would be to reduce the desorption energy of hydrogen [149,151] and to break H-H and Al-H bonds as a result of balanced electron-accepting/back-donation [151].
5. Additional effects. Other effects, such as the formation of mobile species or vacancies, the changes in the Fermi level of reacting species, or the destabilization of Al–H bonds, can also influence the hydrogenation/dehydrogenation reactions [134].

#### 3.2.4. Reactive Mixtures (Composites) with NaAlH_4_

##### Composites of NaAlH_4_-MgH_2_

Composites of NaAlH_4_ and MgH_2_ in several proportions (4:1, 2:1, and 1:1) have been studied in the past years [152,153]. In some cases, catalysts, such as TiF_3_ [154], TiO_2_ [155], or TiH_2_ [156], have been used. The composites in general present four dehydrogenation reactions [152,154]:

NaAlH_4_ + MgH_2_ → NaMgH_3_ +Al + 1.5H_2_ (170–212 °C) (28)

17MgH_2_ + 12Al → Mg_17_Al_12_ + 17H_2_ (280–330 °C) (29)

NaMgH_3_ → NaH + Mg + H_2_ (330–360 °C) (30)

NaH → Na + ½ H_2_ (375 °C and higher) (31)

Only the first three reactions are relevant for hydrogen storage purposes. The reported values of hydrogen released in the first cycle of dehydrogenation ranged between 6.7–7.2 wt.% [152,154,155]. However, these values consider the decomposition of NaH. Prolonged ball milling or the use of catalysts produced a decrement of the activation energy and dehydrogenation temperatures in all steps [152,153,154,155,156]. Nano-confinement in carbon aerogel scaffolds reduced the dehydrogenation steps from four to only two [157]. Regarding the reversibility, up to six hydrogenation/dehydrogenation cycles have been demonstrated when the composite is mixed with carbon nanotubes and graphene nanosheets. In this case, the hydrogen storage level is around 3.5 wt.% at 275 °C and 76 bar [158]. Reaction (28) occurs before NaAlH_4_ decomposes to Na_3_AlH_6_. Thus, a mutual destabilization between NaAlH_4_ and MgH_2_ was proposed as the reaction drive force [152,154]. Ismail et al. mixed MgH_2_ and Na_3_AlH_6_ (4:1) [159]; in this composite, the dehydrogenation pathway is initiated by the following reaction:

Na_3_AlH_6_ + 3MgH_2_ → 3NaMgH_3_ +Al + 3/2 H_2_ (120–250 °C) (32)

The rest of the steps are similar to the reaction sequence (29)–(31).

##### Other Composites with NaAlH_4_

The LiBH_4_-NaAlH_4_ system was studied in two stoichiometric proportions, 1:0.5 and 1:1.15, with theoretical hydrogen storage capacity of 11.9 and 9.8 wt.%, respectively [160]. A metathesis reaction can occur during ball milling or during heating (~95 °C) depending on the amount of reactants and the energetics of the mixing (mortar vs. ball milling) [160]:

LiBH_4_ + NaAlH_4_ → LiAlH_4_ + NaBH_4_ (33)

The first dehydrogenation reaction is the decomposition of LiAlH_4_ to produce Li_3_AlH_6_, Al and H_2_ (~105–110 °C). The dehydrogenation pathway differs according to the excess of initial NaAlH_4_. If an excess of NaAlH_4_ is present, it reacts with Li_3_AlH_6_ to form LiNa_2_AH_6_, LiH, Al, and H_2_ (~200 °C). LiNa_2_AH_6_ decomposes at ~290 °C. Without excess of NaAlH_4_, Li_3_AlH_6_ decomposes at ~180 °C. NaBH_4_ (diffraction peaks) disappear at ~340 °C in both cases. Further heating can lead to the formation of Li-Al alloys and AlB_2_ phases [160]. 

Rehydrogenation was confirmed at ~110 bar hydrogen pressure and 400 °C. The rehydrogenation product was LiBH_4_, as confirmed by in-situ synchrotron radiation powder X-ray diffraction.

#### 3.2.5. Potassium Alanate

KAlH_4_ has an acceptable total hydrogen content of 5.75 wt.% and a reversible hydrogen storage capacity of 4.3 wt.% (through reactions (34) and (35)). These values are comparable to NaAlH_4_ and, additionally, KAlH_4_ does not need a catalyst to undergo re-hydrogenation at a hydrogen pressure as low as 10 bar [161]. KAlH_4_ can be produced by direct synthesis in organic solvent from KH, Al, and hydrogen [21], or in powder form under high pressure of hydrogen (>175 bar) and heating [162], or by mechanical milling, followed by hydrogen exposure [161], or by the reactive mechanical milling in hydrogen atmosphere [163,164], or by the metathesis of NaAlH_4_ or LiAlH_4_ with KCl promoted by ball milling [165].

The dehydrogenation ad re-hydrogenation reactions most “commonly accepted” are [166]:

KAlH_4_ → 1/3 K_3_AlH_6_ + 2/3 Al + H_2_ (~250–330 °C) (34)

1/3K_3_AlH_6_ → KH + 1/3Al + ½ H_2_ (~340 °C) (35)

Global reaction: KAlH_4_ → KH + Al + 3/2H_2_ (36)

A third reaction is the decomposition of KH; however, this reaction is not of interest in hydrogen storage applications. An explanation of “commonly accepted” is required; for KAlH_4_ dehydrogenation and rehydrogenation reactions pathways are still not fully understood. Dehydrogenation pathway involving reactions (34) and (35) are similar to LiAlH_4_ and NaAlH_4_, and it is supported by pressure –composition isotherm (PCI) curves that present two plateaus (1 bar and 10 bar) at 355 °C [166,167]. Additionally, some DFT calculations indicate that K_3_AlH_6_ is sufficiently thermally stable to behave as an intermediary [168]. Santhanam et al. reported the synthesis of K_3_AlH_6_ by 12 h of the mechanical milling of KAlH_4_ and 2KH [169]. However, a number of experimental reports indicate the presence of other reaction intermediaries, such as KAlH_2_ [170], AlH_3_ [171], K*_x_*AlH*_y_* [167], or other phases with partially known crystallography [172]. Some of them were observed during the in-situ synchrotron radiation powder X-ray diffraction experiments; however, they have not been isolated [172]. The controversial results indicate a possible dependency of the dehydrogenation path of KAlH_4_ on the operating conditions, as pointed out by Ares et al. [164]. Additives, such as TiCl_3_ [164,167], or salts, such as NaCl and LiCl (the other product of the ball milling metathesis) [165], could modify the reaction kinetics. 

**Figure 9:**
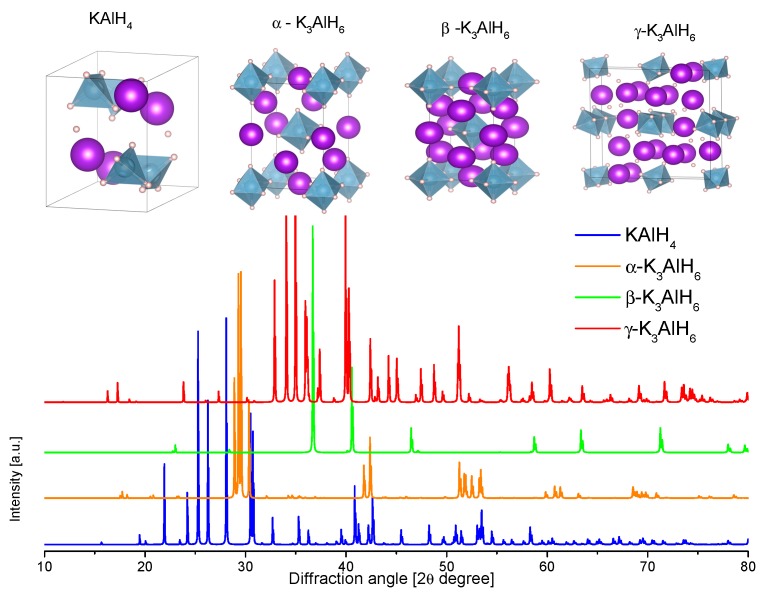
Crystal structure of potassium alanates and their calculated diffraction patterns (λ = Cu_kα1_).

The structure of KAlD_4_ was reported by Hauback et al. in 2005 (Table 4, Figure 9) [173]. KAlD_4_ takes the same structure as BaSO_4_ and KGaD_4_, i.e., the space group *Pnma* [173,174]. The experimental and theoretical studies coincide on a small distortion of the [AlH_4_]^−^ ion from the ideal tetrahedron [173,174]. More interesting is the case of the K_3_AlH_6_ structure; Vajeeston et al. reported three different K_3_AlH_6_ structures according to first-principles studies (Table 4, Figure 9) [175]. The α-K_3_AlH_6_ phase is isostructural with α-Na_3_AlF_6_, and it transforms into the high-pressure structures β-K_3_AlH_6_ and γ-K_3_AlH_6_:

(37) $$\alpha -K_{3}{\mathrm{AlH}}_{6}\;\overset{534\;kbar}{\to }\;\beta -K_{3}{\mathrm{AlH}}_{6}\;\overset{602\;kbar}{\to }\;\gamma -K_{3}{\mathrm{AlH}}_{6}$$

**Figure 10:**
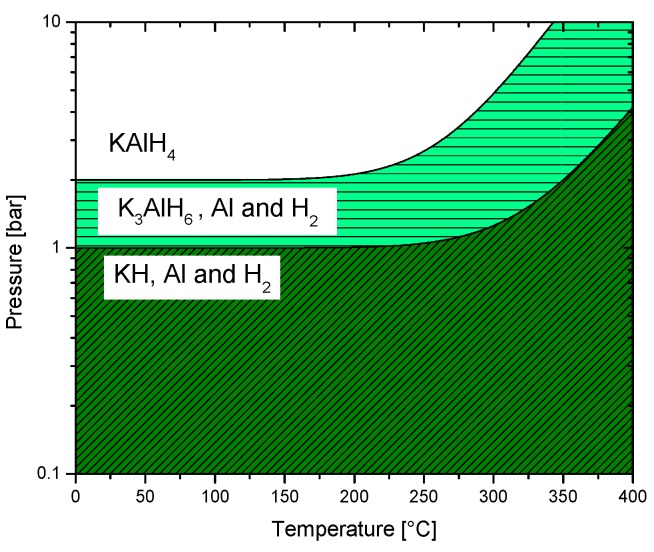
Phase diagram of KAlH_4_, K_3_AlH_6_, and KH + Al. Constructed with data of reference [167] K_3_AlH_6_/KAlH_4_: $\ln\left(\frac{p_{eq}}{p}\right)=-\frac{70\;\mathrm{kJ}\cdot {\mathrm{mol}}^{-1}}{RT}+\frac{130\;J\cdot {\mathrm{mol}}^{-1}\;K^{-1}}{R}$. KH and Al/K_3_AlH_4_: $\ln\left(\frac{p_{eq}}{p}\right)=-\frac{81\;\mathrm{kJ}\cdot {\mathrm{mol}}^{-1}}{RT}+\frac{130\;J\cdot {\mathrm{mol}}^{-1}\;K^{-1}}{R}$.

The experimental dehydrogenation enthalpies for reactions (34) and (35) are 70 ± 2 and 81 ± 2 kJ/mol H_2_, respectively [167]. A phase diagram was generated with these values (Figure 10). In this diagram, the feasibility of hydrogenation at low pressure is evident and it justifies the rehydrogenation without the need for a catalyst or additives. 

#### 3.2.6. Rubidium Alanate

RbAlH_4_ has a hydrogen content of 3.4 wt.%. If this material follows the group 1 tendency regarding dehydrogenation reactions, RbAlH_4_ could reach a 2.5 wt.% of reversible hydrogen storage. Weidenthaler et al. reported the synthesis of RbAlH_4_ from the metals Al, Rb, and with TiCl_3_ as an additive; milling was performed in a hydrogen atmosphere (200 bar) [176]. Adkis et al. reported the synthesis of RbAlH_4_ by the reaction between LiAlH_4_ and metallic Rb [177]. Bestide et al. reported the metathesis between LiAlH_4_ and rubidium halides that are assisted by triethylaluminum (AlEt_3_) in toluene, hexane, and diethyl ether [178]. A stoichiometric reaction (99% product) was almost obtained in the latter work. This reaction yield was explained by the formation of a complex between the halide salts and the triethylaluminum, i.e., a Ziegler-type complex. RbAlH_4_, and the deuterated species were also produced by the metathesis reaction between NaAlH_4_, LiAlH_4_, or LiAlD_4_ with RbCl or RbF promoted by ball milling [176]. RbAlH_4_ or RbAlD_4_ were further heated in an autoclave and then purified [176].

RbAlH_4_ decomposes in two steps at 300 °C and 350 °C (peak temperatures in TG-DCS curves) [176]. However, no complete dehydrogenation and full reversibility have been demonstrated. There is no consensus regarding the dehydrogenation pathway. Weidenthaler et al. proposed that the two dehydrogenation events are related to the formation of RbH plus Al, and the decomposition of RbH, respectively [176]. For its part, Dymova el at. proposed a first decomposition that is associated with the formation of Rb_3_AlH_6_ at 317–334 °C and a second dehydrogenation step by the formation of RbH at 390–417 °C [176,179]. Further confirmation of the dehydrogenation pathway and the formation of Rb_3_AlH_6_ is needed. 

**Figure 11:**
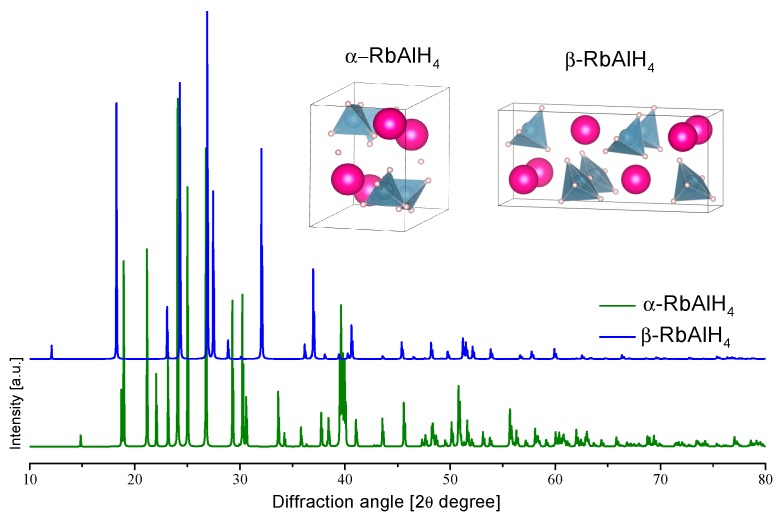
Crystal structure of rubidium alanates and their calculated diffraction patterns (λ = Cu_kα1_).

The structure of RbAlH_4_ was calculated by Vajeeston et al. [180] and then further confirmed by Weidenthaler et al. (Table 5 and Figure 11) [176]. By means of ab-initio calculations, two high-pressure RbAlH_4_ phases are anticipated [181]:

(38) $${\mathrm{RbAlH}}_{4}\;(Pmma)\;\overset{32\;kbar}{\to }\;{\mathrm{RbAlH}}_{4}\;(I41/a)\;\overset{68\;kbar}{\to }\;\mathrm{RbAlH}4_{\;}(\mathrm{Cmc}21)$$

However, no further details regarding the experimental crystallographic data were reported [181]. Ravindran et al. reported the structure of RbAlH_4_ obtained by theoretical calculations. This structure corresponds to a high-pressure phase above ~55 kbar [182]. 

#### 3.2.7. Cesium Alanate

CsAlH_4_ has a hydrogen content of 2.4 wt.%; thus, the interest in CsAlH_4_ is pure chemistry research and is hardly relevant for hydrogen storage. CsAlH_4_ has been prepared by mechanical milling or solvent metathesis of NaAlH_4_ and CsCl, with subsequent purification [183,184]. Previously, Bestide et al. reported the metathesis between LiAlH_4_ and cesium halides assisted by triethylaluminum (AlEt_3_) in toluene, hexane, and diethyl ether [178]. CsAlH_4_ decomposition is marked by four endothermic events [180]:

1. 210–229 °C: polymorphic transition, the material gets an intense yellow color.
2. 280–302: hydrogen evolution due to the proposed reaction:
  3CsAlH_4_ → 2CsH + CsAl_3_H_8_ + H_2_ (39)
3. 454–485 °C: further decomposition reaction of 2CsH + CsAl_3_H_8_:
  2CsH + CsAl_3_H_8_ → 3Cs + 5H_2_ + 3Al (40)
4. 666–672 °C: melting of Al. This reaction pathway does not follow the same decomposition and formation of intermediaries as the rest of the alanates of group 1. In-situ diffraction data is missing for further confirmation of this proposed decomposition pathway. Krech et al. [183] demonstrated a reversible polymorphic transformation between orthorhombic and tetragonal CsAlH_4_; the transformation can be activated by ball-milling or by thermal treatment:

(41) $${\mathrm{CsAlH}}_{4}{\left(o\right)}_{\underset{thermal\;treatment\;at\;200{}^{\circ}C\;}{\leftarrow }}^{\overset{ball-milling\;at\;200\;bar\;H_{2}}{\to }}{\mathrm{CsAlH}}_{4}\left(t\right)$$

**Figure 12:**
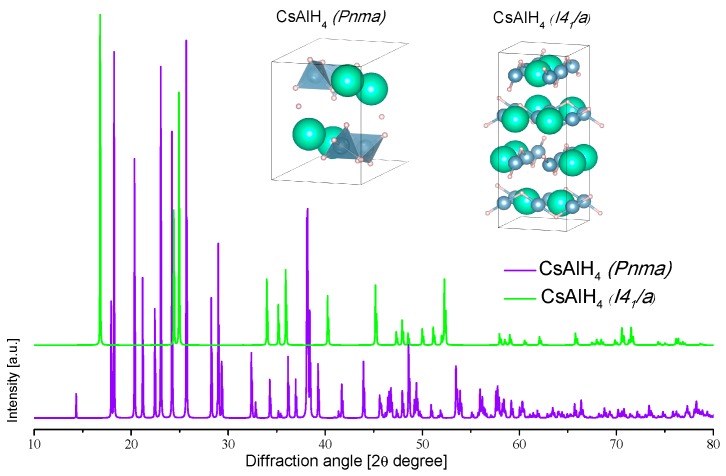
Crystal structure of cesium alanates and their calculated diffraction patterns (λ = Cu_kα1_).

Table 6 lists the collected experimental crystallographic data of cesium alanates (Figure 12). 

### 3.3. Alanates of Group 2

In group 2, in principle, the expected alanates would be M(AlH_4_)_2_ and MAlH_5_. The alanates of group 2 will be discussed in the following sections. 

#### 3.3.1. Beryllium-Alanate

The existence of Be(AlH_4_)_2_ is questionable. Some reviews list the Be(AlH_4_)_2_ phase with a dehydrogenation temperature of 20 °C [186]. The cited reference of these reviews is a book of relatively difficult access [187], whih in turn refers to a series of published works on borohydrides and other boron compounds [188,189]. However, these references dealt with the synthesis of Be(BH_4_)_2_, not Be(AlH_4_)_2_ [188,189]. In 1973, Ashby et al. attempted to produce Be(AlH_4_)_2_ from LiAlH_4_, or NaAlH_4_, and BeCl_2_ in diethyl ether and THF without success [190]. In favor of the existence of Be(AlH_4_)_2_ is the report of Wiberg et al. (1951) [191]. In this work, the reaction between BeCl_2_ and LiAlH_4_ was proposed to produce Be(AlH_4_)_2_ in ether at 20 °C. However, no further details were presented. 

**Figure 13:**
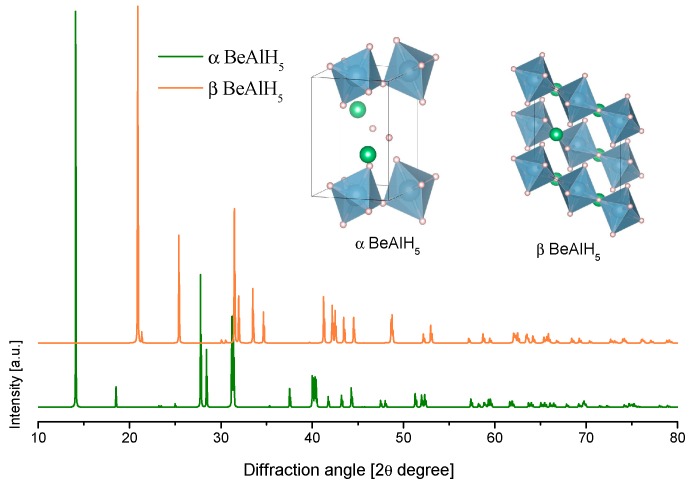
Crystal structure of beryllium alanates and their calculated diffraction patterns (λ = Cu_kα1_).

Only a few theoretical works on BeAlH_5_ have been published. Klaveness et al. reported two calculated structures of BeAlH_5_; the low and high-pressure phases, namely, the α and β phases [192]. However, these calculations were estimated at 0 K, and it was not clear whether BeAlH_5_ could be stable at ambient conditions in that work. Later, Santhosh et al., also by first-principle calculations, found that the α-BeAlH_5_ phase could be stable at ambient (*p* and *T*) conditions [193]. The calculated α-BeAlH_5_ phase consisted of alternating sheets of corner-sharing (AlH_6_) octahedra and chains of corner-sharing (BeH_4_) tetrahedra. On the other hand, the calculated β-BeAlH_5_ phase only consisted of chains of corner-sharing (AlH_6_) octahedra (Table 7 and Figure 13) [192]. 

#### 3.3.2. Magnesium Alanate

Mg(AlH_4_)_2_ has been known since the 1950s [25,27]. At that time, magnesium alanate was synthesized in an organic solvent by the reaction between magnesium hydride and aluminum tri-halides, Equation (6). After almost 50 years, the solid state version of reaction (6) was reported on by Dymova et al. [39] and others [194]. Additionally, roughly at the same time, the metathesis reaction between NaAlH_4_ and MgCl_2_ in organic solvent was reported [30]. In the synthesis that involves organic solvents, the formation of adducts, and the purification (drying without decomposing the alanates), is a frequent problem. Thus, more recently, the metathesis reaction between NaAlH_4_ (or LiAlH_4_) and MgCl_2_, as assisted by mechanical milling, was published and frequently used [43,195].

Mg(AlH_4_)_2_ has a hydrogen content of 9.3 wt.%; however, dehydrogenation studies report values in the range of 6–7 wt.% in the first dehydrogenation step [196]. The most accepted dehydrogenation pathway assumes that Mg(AlH_4_)_2_ decomposes in the temperature range of 110–200 °C, according to the reaction [196,197]:

Mg(AlH_4_)_2_ →MgH_2_ + 2Al + 3H_2_. (42)

Subsequently, further dehydrogenation of MgH_2_ in the presence of Al leads to the formation of Mg-Al compounds of several reported stoichiometries [197,198]. Reports indicate that the dehydrogenation temperature can be reduced by additional milling [199], the addition of materials, such as TiF_4_, TiF_3_ [200], and TiCl_3_ [31], or the reduction of particle size [196,198]. Possibly, the other metathesis product, i.e., LiCl or NaCl, can also produce a change in the dehydrogenation temperatures [195]. Rehydrogenation is partially achieved by the formation of MgH_2_ instead of Mg(AlH_4_)_2_ [31,195,200]. However, Gremaud et al. reported the formation of Mg(AlH_4_)_2_ at 1 bar, and 100 °C from thin films of Mg-Al covered with a thin layer of Ti [201]. 

**Figure 14:**
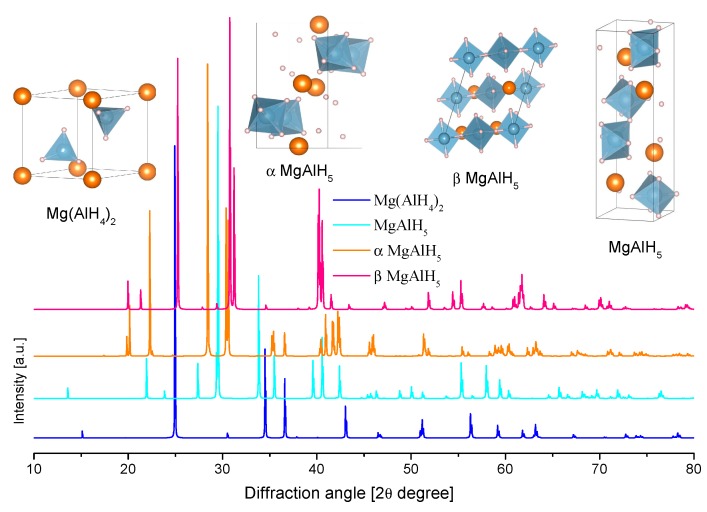
Crystal structure of magnesium alanates and their calculated diffraction patterns (λ = Cu_kα1_).

The crystal structure of Mg(AlH_4_)_2_ was determined by a combination of X-ray and neutron diffraction at 295 K (Table 8 and Figure 14.) [202]. The crystallographic information is consistent with other experimental and theoretical reports [203,204,205]. The structure consists of [AlH_4_]^−^ tetrahedra that formed double layers that were perpendicular to the **c** axis of the trigonal cell and alternating with Mg layers (Figure 14) [205].

MgAlH_5_ was originally proposed by Dymova et al. as a reaction intermediary of the decomposition of Mg(AlH_4_)_2_ [39]. However, further experimental reports did not confirm this. Other elements of group 2 (M) indeed form MAlH_5_ compounds. On the other hand, theoretical calculations indicate the possible crystals structures of MgAlH_5_ (Table 8). 

The few available kinetic studies indicate that the dehydrogenation reaction is ruled by the diffusion of MgH_2_, Al, or hydrogen in the TiF_4_ doped samples [197,208]. In any case, the activation energy of dehydrogenation reaction (42) is high: 117.5 [206]–123 [197] kJ/mol. Theoretical studies indicate that Mg(AlH_4_)_2_ is metastable at room temperature with a formation enthalpy of −21 kJ/mol H_2_ [209]. By ab-initio calculations, Spanò et al. determined, that the dehydrogenation temperature at atmospheric pressure must be 111 °C [210]. Thus, despite the interesting high hydrogen content and low dehydrogenation temperatures, Mg(AlH_4_)_2_ can be classified as a one-way hydrogen storage material. 

#### 3.3.3. Reactive Mixtures (Composites) with Mg(AlH_4_)_2_

Few examples of the composites of Mg(AlH_4_)_2_ were found during the preparation of this review; they are, in summary: Mg(AlH_4_)_2_-NaAlH_4_ [211,212], Mg(AlH_4_)_2_-MgH_2_ [213], Mg(AlH_4_)_2_-LiBH_4_ [214,215], and Mg(AlH_4_)_2_-Ca(BH_4_)_2_ [216]. The original reports include several stoichiometries and preparation procedures. However, they have the reduction of dehydrogenation temperature as compared with the individual components and a relatively high amount of hydrogen released during the first dehydrogenation step in common. In many cases, the role of Mg(AlH_4_)_2_ is classified as a catalyst of the other components of the mixture. Usually, the first dehydrogenation step corresponds to the decomposition of Mg(AlH_4_)_2_, i.e., reaction (42). Afterwards, MgH_2_ reacts with other components of the mixture. For example, in the Mg(AlH_4_)_2_-NaAlH_4_ composite, NaMgH_3_ was formed, and the proposed reaction is [211]:

2Na_3_AlH_6_ + 6MgH_2_ → 6NaMgH_3_ + 2Al + 3H_2_ (43)

Further decomposition of NaMgH_3_ in the presence of Al leads to the formation of Mg-Al alloys.

For the Mg(AlH_4_)_2_-LiBH_4_ composite, Liu et al. proposed as a second step the formation of Mg_2_Al_3_ from the reaction of MgH_2_ and Al. Subsequently, Mg_2_Al_3_ reacts with LiBH_4_ [214]:

6LiBH_4_ + 0.5Mg_2_Al_3_ + 0.5Al → 6LiH + MgAlB_4_ +AlB_2_ + 9H_2_ (44)

The formation of MgAlB_4_ was also proposed by Pang et al. [215]. Two main drawbacks are observed for the composites of Mg(AlH_4_)_2_:

1. Despite the reduction in dehydrogenation temperatures, the “ideal” dehydrogenation temperature—compatible with PEM fuel cells—is not attained.
2. Re-hydrogenation is only partially achieved through the formation of MgH_2_, not Mg(AlH_4_)_2_.

#### 3.3.4. Calcium Alanate

Reports on the synthesis of Ca(AlH_4_)_2_ dates from the 1950s [28]; back then, the synthesis was performed in an organic solvent by the reaction between CaH_2_ and AlCl_3_ [28] or AlBr_3_ [217]. As other alanates, the synthesis of Ca(AlH_4_)_2_ evolved towards the metathesis reaction between NaAlH_4_ or LiAlH_4_ and CaCl_2_ in an organic solvent [31], to finally take advantage of the use of mechanical milling to perform direct or metathesis synthesis. As for other alanates, the synthesis in organic solvents, such as THF, produced adducts of complicated purification without decomposition of the alanate. Thus, the synthesis that is assisted by mechanical milling is nowadays popular [218]. 

Calcium alanate has a total hydrogen content of 7.9 wt.%. Its complete decomposition occurs in four steps; the first two steps liberate 5.2–5.9 wt.% hydrogen of the theoretical 7.15 wt.% [165,219,220]. The dehydrogenation reactions are [220]:

Ca(AlH_4_)_2_ → CaAlH_5_ + Al + 3/2H_2_ (100–160° C) (45)

CaAlH_5_ → CaH_2_ + Al + 3/2H_2_ (220–270 °C) (46)

CaH_2_ + 4Al → Al_4_Ca + H_2_ (~350 °C) (47)

CaH_2_ + Al_4_Ca → 2Al_2_Ca + H_2_ (~400 °C) (48)

Adding TiF_3_ led to a decrease in the activation energy and the dehydrogenation temperature [219]. Li et al. suggest that the F atoms from TiF_3_ substitutes H atoms in Ca(AlH_4_)_2_ and that TiF_3_ initiates the decomposition of calcium alanate [219]:

(49) $$\mathrm{Ca}({\mathrm{AlH}}_{4}{)}_{2}\;+\;\frac{x}{3}{\mathrm{TiF}}_{3}\;\to \;{\mathrm{CaAlF}}_{x}H_{5-x}\;+\;\mathrm{Al}\;+\;\frac{x}{3}\mathrm{Ti}\;+\;\frac{x+3}{2}H_{2}.\;$$

**Figure 15:**
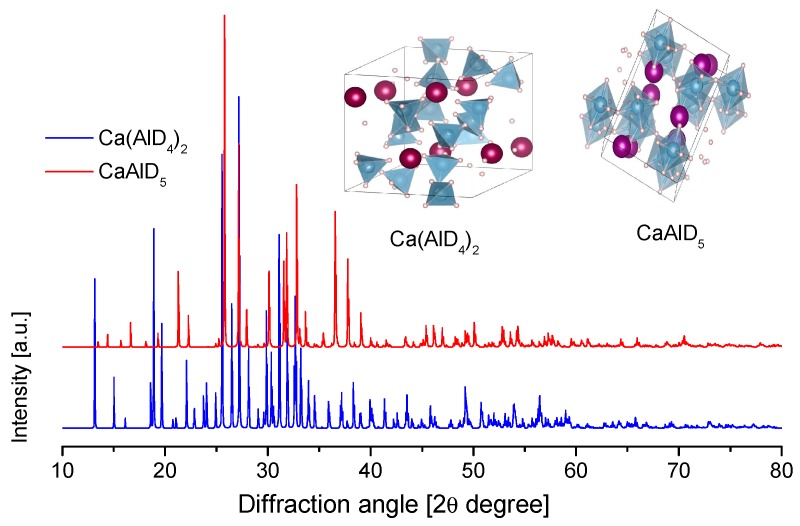
Crystal structure of calcium alanates and their calculated diffraction patterns (λ = Cu_kα1_).

The crystal structure of Ca(AlH_4_)_2_ and CaAlH_5_ were determined in 2009 (Table 9 and Figure 15) [221]. However, theoretical predictions and partial experimental reports were published as early as 2005–2006 [222,223,224]. Ca(AlD_4_)_2_ takes an orthorhombic Ca(BF_4_)_2_-type structure [221]. Meanwhile, the crystal structure of CaAlD_5_ was found to be a monoclinic α-SrAlF_5_-type structure [221]. CaAlH_5_ consists of corner-sharing (AlH_6_) octahedra [224].

**Figure 16:**
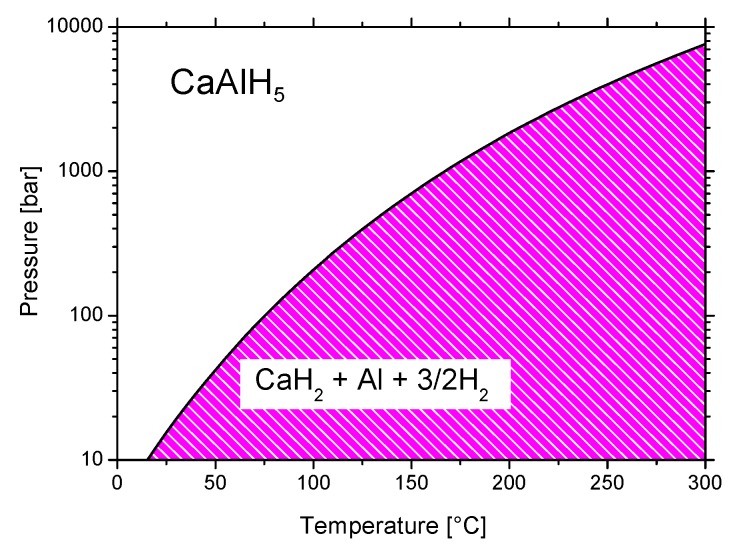
Phase diagram of CaAlH_5_, CaH_2_, and Al. CaH_2_ and Al/CaAlH_5_: $\ln\left(\frac{p_{eq}}{p}\right)=-\frac{32\;\mathrm{kJ}\cdot {\mathrm{mol}}^{-1}}{RT}+\frac{130\;J\cdot {\mathrm{mol}}^{-1}\;K^{-1}}{R}$ [224].

Ca(AlH_4_)_2_ decomposition is slightly exothermic [224], with the enthalpy of reaction (45) being about −7 [220] to −9 kJ/mol H_2_ [224]. The second dehydrogenation step (reaction (46)) is endothermic with an enthalpy of 32 kJ/mol H_2_ [224]. The reversibility of Equations (45) and (46) was not reported. The enthalpy values indicate that the first reaction is not suitable for hydrogen storage for mobile applications. However, the second reaction, in principle, could be suitable for mobile hydrogen storage. The enthalpy value of reaction (46) was used to generate the phase diagram of Figure 16. The diagram indicates that CaAlH_5_ could be produced at temperatures and pressures that are compatible with fuel cells, perhaps with the help of a proper catalyst. In this scenario, the reversible hydrogen storage capacity would be 4.19 wt.%. 

#### 3.3.5. Reactive Mixtures (Composites) with Ca(AlH_4_)_2_

Scarce examples of reactive mixtures with Ca(AlH_4_)_2_ were found during the redaction of this review. One of them was the mixture of LiBH_4_ and Ca(AlH_4_)_2_, giving the best results with a molar ratio of 6:1, respectively [225]. In that system, the released hydrogen was 8.2 wt.% up to 450 °C. Reactions (45) and (46) initiate the dehydrogenation pathway. Subsequently, LiBH_4_ reacts as: [225] 

8LiBH_4_ + CaH_2_ + Al → CaB_6_ + AlB_2_ + 8LiH + 13H_2_. (50)

The last step is the reaction (47) of the remaining materials. Rehydrogenation was demonstrated at 450 °C and 40 bar to produce LiBH_4_ and Ca(BH_4_)_2_ and 4.5 wt.% hydrogen storage. 

Hanada et al. reported the dehydrogenation of Ca(AlH_4_)_2_ + Si, Ca(AlH_4_)_2_ + 2MgH_2_, Ca(AlH_4_)_2_ + 2LiH, and Ca(AlH_4_)_2_ + 2LiNH_2_ mixtures that were produced by manual or ball milling [226]. In their work, Ca(AlH_4_)_2_ was produced by a metathesis reaction and it was used without purifying, i.e., with the load of NaCl. The weight losses were 6.1 wt.% for Ca(AlH_4_)_2_ + 2MgH_2_ and 5.5 wt.% for manually milled Ca(AlH_4_)_2_ + 2LiNH_2_. These values were reported without taking the load of NaCl into account. The rest of the hydrogen release values were not clearly specified. For the Ca(AlH_4_)_2_ + Si mixture, the first two reactions are the usual Ca(AlH_4_)_2_ dehydrogenation reactions, Si does not react with CaH_2_ or Ca-containing phases [226]. For the Ca(AlH_4_)_2_ + 2MgH_2_ mixture, after the usual first two dehydrogenation reactions, MgH_2_ decomposes at around 270–350 °C and then reacts with Al to form Al_12_Mg_17_ [226]. Alapati et al., by means of first-principle calculations, arrived at the same reactions, plus a high-temperature reaction [227]:

6CaH_2_ + Al_12_Mg_17_ → 17Mg + 6Al_2_Ca + 6H_2_ (~600 °C) (51)

For the Ca(AlH_4_)_2_ + 2LiH mixture, CaAlH_5_ is generated during ball milling due to the solid-state reaction between Ca(AlH_4_)_2_ and LiH [226]. Meanwhile, Li_3_AlH_6_ appears after heating to 150 °C under 3 bar of He. Subsequently, at 250 °C, the CaH_2_ and Al phases arise and Li_3_AlH_6_ disappears [226]. For the Ca(AlH_4_)_2_ + 2LiNH_2_ mixture, a reaction of decomposition of Ca(AlH_4_)_2_ with LiNH_2_ occurs during ball milling. A similar hand-milled mixture produced the same two dehydrogenation reactions of Ca(AlH_4_)_2_, plus the reaction:

CaH_2_ + 2LiNH_2_ → Li_2_NH + CaNH + 2H_2_ (52)

The last reaction is reported to simultaneously occur with Equation (47) in this system [226]. The re-hydrogenation reactions are not reported. 

#### 3.3.6. Strontium Alanates

The system Sr-Al-H presents a richness of chemistry and compounds. Here, we present the most representative characteristics reported for them. Sr(AlH_4_)_2_ has a hydrogen content of 5.3 wt%. It was first produced by the reaction between SrH_2_ and AlH_3_ by mechanochemical activation in 2000 [228]. After that, Sr(AlH_4_)_2_ was produced by the metathesis reaction between SrCl_2_ and 2NaAlH_4_, being assisted by mechanical milling [44]. The decomposition of Sr(AlH_4_)_2_ initiated at about 130 °C. Subsequently, a second dehydrogenation step occurred at about 240 °C to achieve a total of 2.1 wt.% of released hydrogen with both reactions (0.8 and 1.3 wt.%, respectively, including the NaCl load) [44]. SrAlH_5_ (4.21 wt.% of total hydrogen content) is proposed as a reaction intermediary of the decomposition of Sr(AlH_4_)_2_ [192,228]:

2Sr(AlH_4_)_2_ → 2SrAlH_5_ + 2Al + 3H_2_ (145–165 °C) (53)

2SrAlH_5_ → 2SrH_2_ + 2Al + 3H_2_ (220–320 °C) (54)

2SrH_2_ + 4Al → Al_4_Sr + SrH_2_ + H_2_ (355–390 °C) (55)

SrH_2_ → Sr + H_2_ (890–950 °C) (56)

Partial rehydrogenation was achieved by (re)milling at high hydrogen pressure (300 bar). Further dehydrogenation demonstrated a drastic reduction of the hydrogen release (about 0.8 wt.%) [44]. 

**Figure 17:**
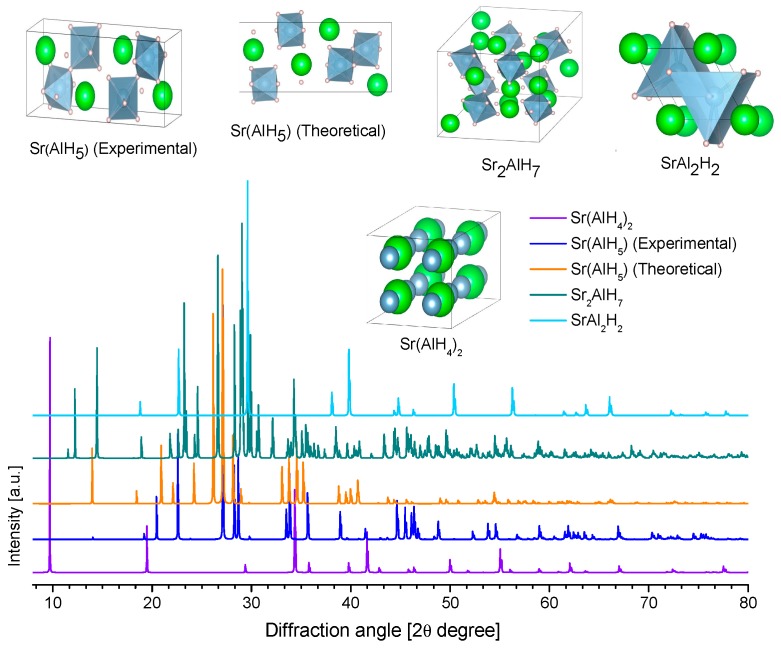
Crystal structure of strontium alanates and their calculated diffraction patterns (λ = Cu_kα1_).

SrAlD_5_ was produced by the mechanical milling of SrD_2_ and AlD_3_ and further heating at 154 °C for 1 h in Ar [229]. SrAlD_5_ was studied by synchrotron and neutron diffraction in detail; the resolved structure consists of (AlD_6_) octahedra that share corner D atom forming a chain (Figure 17) [229]. This was the first complete experimental report on the crystallography of SrAlD_5_ (Table 10). Previously, the partial crystal structure (no H positions given) [44] and first-principle crystallographic data of SrAlH_5_ were reported [192]. The calculated and the experimental data appreciably differ (Figure 17). 

Sr_2_AlH_7_ (3.37 wt.% of hydrogen content) was produced by the mechanical milling of SrAl_2_ and further hydrogenation at 70 bar and 270 °C for ten days. The arc melting of Sr and Al previously prepared SrAl_2_ [230]. Zhang et al. reported that the crystal structure of Sr_2_AlD_7_ consisted of isolated (AlD_6_) units and one-dimensional chains of edge-sharing (DSr_4_) tetrahedra [230]. 

The proposed formation pathway is [231,232,233]:

SrAl_2_ + H_2_ → SrAl_2_H_2_ (190 °C, 50 bar) (57)

4SrAl_2_H_2_ + 3H_2_ → 2Sr_2_AlH_7_ + 6Al (240 °C, 70 bar) (58)

The milling of SrAl_2_ in hydrogen atmosphere led to the formation of SrH_2_ and Al. The milled materials were further hydrogenated at 260 °C (no pressure indicated) for two days to give Sr_2_AlH_7_ [231]:

4SrH_2_ + 2Al +3H_2_ → 2Sr_2_AlH_7._ (59)

On the other hand, Sr_2_AlH_7_ decomposes to SrH_2_, Al, and H_2_ at 290 °C [231,232]. However, attempts to hydrogenate mixtures of 4SrH_2_ + 2Al (70 bar, 260 °C, two days) did not succeed. In this last case, stearic acid was used as a process control agent (PCA) to avoid the cold welding of Al during mechanical milling. Possibly, another PCA might lead to successful hydrogenation. Unfortunately, the dehydrogenation curves of Sr_2_AlH_7_ were not presented in these studies. 

Table 10 lists the collected crystallographic information of Sr-Al-H compounds.

#### 3.3.7. Barium Alanates

**Figure 18:**
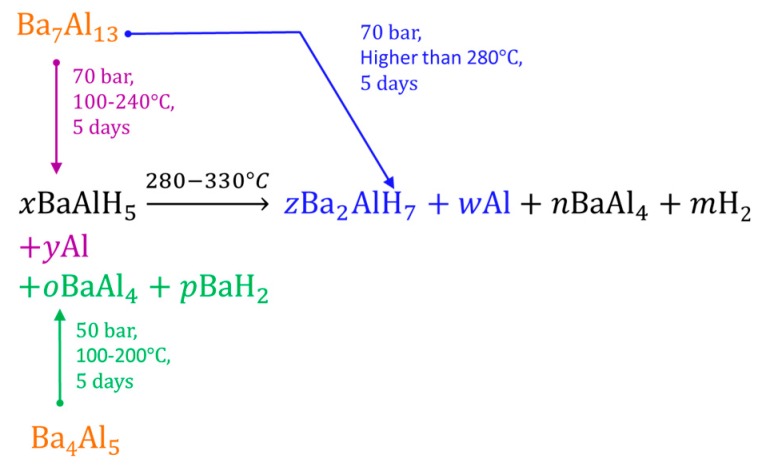
Production of BaAlH_4_ or B_2_AlH_7_ from hydrogenation of Ba_7_Al_13_ or Ba_4_Al_5_.

For Barium, two barium-aluminum-hydride compounds have been reported: BaAlH_5_ (2.97 wt.% hydrogen content) and Ba_2_AlH_7_ (2.28 wt.% hydrogen content). They have been prepared from Ba_7_Al_13_ or Ba_4_Al_5_ alloys, followed by ball-milling and several days in hydrogenation conditions (70 bar, ~200 °C). The Ba_7_Al_13_ and Ba_4_Al_5_ alloys were previously prepared by arc melting [234,235,236]. Alternatively, the reactive ball milling of the mixture of BaH_2_ and Al can produce the BaAlH_5_ and Ba_2_AlH_7_ [237]. The formation of BaAlH_5_ or Ba_2_AlH_7_ can be directed by the choice of precursor or by the selection of the temperature (Figure 18) [234,235,236]. BaAlH_5_ and Al were produced by the hydrogenation of Ba_7_Al_13_ (dark pink reaction, Figure 18). Meanwhile, BaAlH_5_, BaAl_4_, and BaH_2_ were produced by the hydrogenation of Ba_4_Al_5_ (green reaction, Figure 18). The further heating of BaAlH_5_ (black reaction, Figure 18) or high-temperature synthesis from Ba_7_Al_13_ (blue reaction, Figure 18) can produce Ba_2_AlH_7_ along with some by-products [234,235,236].

Liu et al. reported a clear effect of the initial stoichiometry of the mixture on the product when a mixture of BaH_2_ and Al was used as the precursor. The 2:1 and 1:1 mixtures directed the product to Ba_2_AlH_7_. Meanwhile, a 1:2 mixture directed the product to BaAlH_5_ [237]. However, none of the mixtures produced a complete reaction. 

Liu et al. proposed the following reactions for the decomposition of BaAlH_5_ and Ba_2_AlH_7_ [236]:

5BaAlH_5_ → Ba_2_AlH_7_ + 2BaH_2_ + BaAl_4_ + 7H_2_ (280 °C, Argon) (60)

4BaAlH_5_ → 3BaH_2_ + BaAl_4_ + 7H_2_ (350 °C, Vacuum) (61)

4Ba_2_AlH_7_ → 7BaH_2_ + BaAl_4_ + 7H_2_ (350 °C, Vacuum) (62)

**Figure 19:**
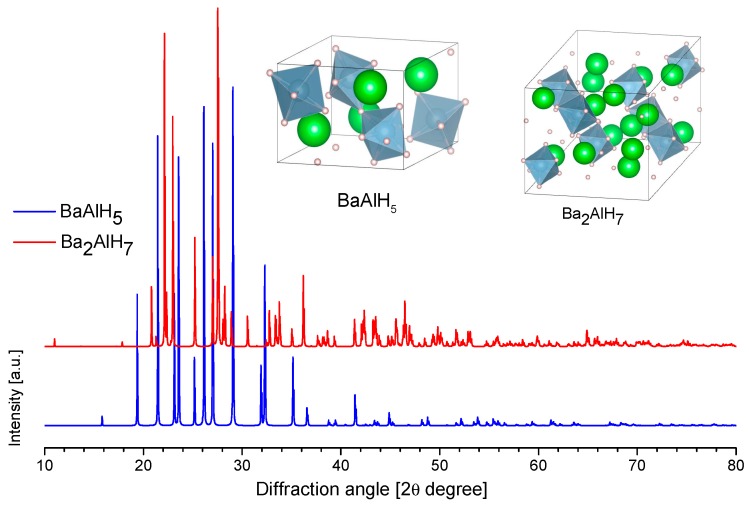
Crystal structure of barium alanates and their calculated diffraction patterns (λ = Cu_kα1_).

Table 11 and Figure 19 present the crystal structures of the barium-aluminum hydrides. The crystal structure of BaAlH_5_ is composed of corner-sharing (AlH_6_) octahedra that form zigzag chains along the crystallographic *c* axis [207]. Meanwhile, Ba_2_AlD_7_ is composed of isolated (AlD_6_) octahedra and infinite one-dimensional chains of edge-sharing (DBa_4_) tetrahedra [235]. 

### 3.4. Alanates of Transition Metals

The alanates of the transition metals date from the 1950s–1960s. Although most of them have decomposition temperatures too low for hydrogen storage purposes, some of them can be of interest. However, almost all of the reported materials have been poorly characterized. Normally, the old reports did not present the basic characterization of materials, for example, X-ray diffraction or infrared spectroscopy. On the other hand, some of them have only been theoretically discussed. In the following sections, the most important (experimental and/or theoretical) characteristics of this family of alanates are presented. 

#### 3.4.1. Scandium Alanate

Charkin et al., in a theoretical study, proposed the decomposition of a hypothetical Sc(AlH_4_)_3_ to provide the following products: a) HSc(AlH_4_)_2_ + AlH_3_, b) H_2_Sc(AlH_4_) + 2AlH_3_, or c) ScH_3_ + 3AlH_3_. This latter route will give the highest dissociation energy [238]. Sc(AlH_4_)_3_ deserves more research to estimate, for example, formation energy or crystal structure. Experimentally, Sc(AlH_4_)_3_ has not been synthesized, despite a possible and interesting 8.7 wt.% of hydrogen content. 

#### 3.4.2. Yttrium Alanate

Y(AlH_4_)_3_ was first described by Kost et al. in 1978 [239]. Later, in 2017, Cao et al. demonstrated the partial reversibility of hydrogen storage [240]. Y(AlH_4_)_3_ has a hydrogen content of 6.6 wt.% and it can be produced by the metathesis reaction between 3LiAlH_4_ + YCl_3_ [240]. 

Kost et al. reported the beginning of decomposition of Y(AlH_4_)_3_ at 50 °C [239]. However, they did not present additional details. Cao et al., based on different characterization techniques, proposed that the decomposition of Y(AlH_4_)_3_ occurs as:

Y(AlH_4_)_3_ → YAlH_6_ + 2Al +3H_2_ (80–170 °C) (63)

YAlH_6_ → YH_3_ + Al + 1.5H_2_ (170–250 °C) (64)

YH_3_ → YH_2_ + 0.5H_2_ (250–350 °C) (65)

YH_2_ + 3Al → YAl_3_ + H_2_ (>350 °C) (66)

In reaction (63), at 140 °C, 3.4 wt.% of hydrogen was released. 2.6 wt.% of hydrogen was re-adsorbed at 145 °C and 100 bar. However, no direct hydrogenation from YH_3_ +Al at 145 °C and 100 bar occurred [240]. Y(AlH_4_)_3_, and YAlH_6_ are reported as amorphous materials [240]. However, no direct evidence of YAlH_6_ was presented [240]; thus, further characterizations of these materials are needed. 

#### 3.4.3. Titanium Alanate

Wiberg et al. reported the formation of Ti(AlH_4_)_4_ (11.1 wt.% hydrogen content) in 1951 [14]. The synthetic route was the metathesis reaction between TiCl_4_ and LiAlH_4_ in ether at −110 °C [14]. Later, in 1975, Kost et al. reported a similar synthesis while using LiAlH_4_ and TiBr_4_ or TiCl_4_. The product was separated from the solution in a filter cooled with dry ice [241]. The reported stoichiometries indicted that the metathesis reaction was not completed or that partial substitution of Cl^−^ by [AlH_4_]^−^ was achieved [241]. Wiberg reported that Ti(AlH_4_)_4_ was decomposed at −85 °C [14]; for its part, Kost reported the evolution of “two g-atom of H per g-atom of Ti” at −70 °C [241]. The decomposition of Ti(AlH_4_)_4_ was proposed as [241]:

Ti(AlH_4_)_4_ → TiH_2_ + 4AlH_3_ + H_2._ (67)

Further decomposition of AlH_3_ was observed at 110 °C [241]. No more characteristics of this material have been reported. However, Ti(AlH_4_)_4_ can be a very interesting material in regards to its hydrogen content, perhaps tailoring the dehydrogenation temperature with some structural or chemical modification could be explored. Another point to discuss is that Ti can work in other oxidation states besides Ti^4+^; for example, Ti^3+^ or Ti^2+^. The Ti^3+^ and Ti^2+^ compounds are generally more stable than the Ti^4+^ compounds, i.e., the liquid and volatile TiCl_4_ versus solid TiF_3_ or TiCl_2_. Alternatively, other compositions of Ti-Al alloys or intermetallics could be explored. For example, Ramzan et al. explored employing DFT calculations, the structural stability, and other properties of Ti_4_AlH_3_ and Ti_3_AlH_2_ phases [242]. Maeland et al., some time ago, reported the reversible hydrogenation of Ti_3_Al at 9.2 bar of deuterium pressure and 200 °C to form Ti_3_AlD*_x_* (*x* = 5.9–8) [243]. 

#### 3.4.4. Zirconium Alanate

The first report on Zr(AlH_4_)_4_ was the work of Reid et al. in 1957 [13]. Zr(AlH_4_)_4_ (7.49 wt.% hydrogen content) was produced by the metathesis reaction between Zr(BH_4_)_4_ and LiAlH_4_ in ether solution and He atmosphere [13]. Zr(BH_4_)_4_ was formerly prepared by metathesis of LiBH_4_ and ZrCl_4_ [13]. In 2008, Zr(AlH_4_)_4_ was produced by the reaction between LiAlH_4_ and ZrCl_4_ in ether solution [244]. No clear indication of the reaction temperature was found in this work. No reports regarding the characteristics of dehydrogenation or on the characterization of this material were found. Other compositions of the Zr-Al-H system deserve further research; for example, Matsubara et al. achieved the hydrogenation of the intermetallic Zr_3_Al to give Zr_3_AlH_4_ [245]. 

#### 3.4.5. Vanadium Alanate

Charkin et al. also proposed the decomposition of a hypothetical V(AlH_4_)_3_ to provide the following products: (a) HV(AlH_4_)_2_ + AlH_3_, (b)H_2_V(AlH_4_) + 2AlH_3_, or (c) VH_3_ + 3AlH_3_ [238]. Experimental confirmation of the existence of V(AlH_4_)_3_ is missing.

#### 3.4.6. Niobium Alanates

Wiberg et al., in 1965, reported the reaction between NbCl_5_ and LiAlH_4_ in several proportions and temperatures in ether at low temperature [246]. Wiberg et al. concluded that the products were a function of the temperature and the excess of LiAlH_4_ used; the first family of products was [246]:

(68) $${\mathrm{NbCl}}_{5}+5{\mathrm{LiAlH}}_{4}\;\to \;\mathrm{Nb}({\mathrm{AlH}}_{4}){}_{n}+(5\;-\;n){\mathrm{AlH}}_{3}+\frac{5-n}{2}H_{2}+5\mathrm{LiCl},$$

when *n* = 3.5 at −70 °C the product was Nb_2_(AlH_4_)_7_, for *n* = 3.0 at −40 °C the product was Nb_2_(AlH_4_)_6_, and for *n* = 2.5 at 20 °C the product was Nb_2_(AlH_4_)_5_. 

The other family of products was:

(69) $${\mathrm{NbCl}}_{5}+(5+m){\mathrm{LiAlH}}_{4}\;\to \;{\mathrm{Li}}_{m}\mathrm{Nb}({\mathrm{AlH}}_{4}){}_{n+m}+(5\;-\;n){\mathrm{AlH}}_{3}+\frac{5-n}{2}H_{2}+5\mathrm{LiCl},$$

LiNb_2_(AlH_4_)_7_ was formed at −70 °C; meanwhile, LiNb_2_(AlH_4_)_5_ and LiNb(AlH_4_)_3_ were formed at 25 °C [246]. Wiberg et al. wonderfully described the synthesis procedure and the changes in the color that are associated with each Nb or LiNb- alanates. However, a detailed characterization is needed, particularly the characterization of the material obtained at room temperature Nb_2_(AlH_4_)_5_ (5.9 wt.% hydrogen content).

#### 3.4.7. Tantalum Alanates

TaH_2_(AlH_4_)_2_ was reported by Kost et al. in 1978 [239]. The compound has a hydrogen content of 4.11 wt.%. It was produced in cold ether by the reaction between LiH, Al and a metal halide. Kost et al. reported that TaH_2_(AlH_4_)_2_ is a red powder that decomposes at 130 °C. TaH_2_(AlH_4_)_2_ and AlH_3_ are the decomposition products of a very unstable Ta(AlH_4_)_n_ [239].

#### 3.4.8. Manganese Alanate

The reports on Mn(AlH_4_)_2_ are rather diffuse, as in the case of Be(AlH_4_)_2_. The first compilation where Mn(AlH_4_)_2_ appeared, is the book of Mackay [187]. In that book, Mn(AlH_4_)_2_ was reported to be prepared from a halide complex (no mention of which halide) and LiAlH_4_ in Et_2_O, and to decompose at 25 °C. The book refers, in turn, to two reports of Monnier et al. [247,248]. No further reports on Mn(AlH_4_)_2_ were found. Mn(AlH_4_)_2_ would have a hydrogen content of 6.89 wt.%. 

#### 3.4.9. Iron Alanate

Fe(AlH_4_)_2_ can be an interesting material for hydrogen storage, due to the 6.84 wt.% of hydrogen content. However, contradictory reports on the decomposition temperature are published. In favor of the near-room temperature stability of Fe(AlH_4_)_2_ is the report of Neumaier et al. [249]. Fe(AlH_4_)_2_ was prepared by means of metathesis of FeCl_3_ + 3LiAlH_4_ in ether at low temperature (−116 °C) [249]. Once formed, the iron easily decomposed. Neumaier et al. presented a *p*-T diagram of the decomposition reaction; around 20 °C a continuous partial decomposition was observed. Meanwhile, a fast decomposition was observed at 90–100 °C. Two comments can be mentioned: (1) The quantity of released hydrogen was not reported despite a detailed thermolysis study being presented. (2) The fast decomposition at 90–100 °C is near to the temperature of α-, and α’-alane decomposition [186], which is one by-product of iron alanate formation. This leave doubts about who is decomposing Fe(AlH_4_)_2_ or AlH_3_. Despite that, Neumaier et al. considered Fe(AlH_4_)_2_ to be stable at room temperature. The proposed reactions of formation and decomposition are [249]:

FeCl_3_ + 3LiAlH_4_ → Fe(AlH_4_)_2_ +AlH_3_ + 3LiCl +0.5H_2_, (70)

Fe(AlH_4_)_2_ → Fe + 2Al + 4H_2_ (71)

Against the near-room temperature stability of Fe(AlH_4_)_2_ is the report of Schaeffer et al. [250]. They also produced Fe(AlH_4_)_2_ by means of metathesis of FeCl_3_ and an excess of LiAlH_4_. However, Schaeffer et al. considered Fe(AlH_4_)_2_ to be unstable at room temperature. 

#### 3.4.10. Copper Alanate

CuAlH_4_ (4.2 wt.% hydrogen content) was reported as a product of the reaction between CuI and LiAlH_4_ in ether at −78 °C by Ashby et al. [251]. CuAlH_4_ is unstable and it reacts quickly, with the proposed product being Cu_3_AlH_6_ [251]:

CuAlH_4_ → CuH + AlH_3_ (72)

2CuH + CuAlH_4_ → Cu_3_AlH_6_ (73)

Both of the alanates decomposed with a slight heating. Wiberg et al. reported that the reaction between CuI and LiAlH_4_ in pyridine at room temperature did not produce Cu-alanates; it produced LiI, AlI_3_, and CuH [252]. 

#### 3.4.11. Silver Alanate

AgAlH_4_ (2.9 wt.% hydrogen content) was produced by the following reaction in ether at −80 °C [253]:

AgClO_4_ + LiAlH_4_ → AgAlH_4_ +LiClO_4_ (74)

AgAlH_4_ decomposed at −50 °C to the elements Ag, Al, and H_2_ [253].

#### 3.4.12. Zinc Alanate

Zhizhin et al. (and references wherein) summarized the production of ZnH_2_; one of the reactions is [254]:

2LiAlH_4_ + ZnI_2_ → 2LiI + 2AlH_3_ + ZnH_2_ (75)

However, depending on the reaction conditions (solvent composition, mainly), admixtures of Zn-AlH_4_ can be present:

(76) $${\mathrm{LiAlH}}_{4}\;\overset{{\mathrm{ZnI}}_{2}}{\to }\;\mathrm{Zn}({\mathrm{AlH}}_{4}{)}_{2}\;\overset{{\mathrm{ZnI}}_{2},{\mathrm{LiAlH}}_{4}}{\to }\;\mathrm{Zn}[{\mathrm{ZnI}}_{2}({\mathrm{AlH}}_{4}{)}_{2}]\;\overset{{\mathrm{ZnI}}_{2},{\mathrm{LiAlH}}_{4}}{\to }\;\mathrm{Zn}[{\mathrm{ZnI}}_{3}({\mathrm{AlH}}_{4})]$$

### 3.5. Alanates of the Main Group

As in the case of transition metals alanates, the alanates of the main group elements are scarce, with most of them being unstable, even at low temperatures. 

#### 3.5.1. Gallium Alanate

Ga(AlH_4_)_3_ (7.4 wt.% hydrogen content) was produced by the reaction between GaCl_3_ and LiAlH_4_ in ether at 0 °C [255,256]:

GaCl_3_ + 3LiAlH_4_ → Ga(AlH_4_)_3_ + 3LiCl (77)

However, at 35 °C, the Ga(AlH_4_)_3_ decomposes into GaH_3_ and AlH_3_ [256]. 

#### 3.5.2. Indium Alanate

In(AlH_4_)_3_ (5.8 wt.% hydrogen content) was produced by the reaction between InCl_3_ and LiAlH_4_ in ether at −70 °C [256]:

InCl_3_ + 3LiAlH_4_ → In(AlH_4_)_3_ + 3LiCl (78)

The In(AlH_4_)_3_ decomposed at −40 °C. However, in a similar reaction at room temperature:

InCl_3_ + LiAlH_4_ → InCl_2_(AlH_4_) + LiCl, (79)

the product InCl_2_(AlH_4_) is stable up to 100 °C [256]. 

#### 3.5.3. Thallium Alanate

The synthesis of TlAlH_4_ was reported in 1967, the reaction was performed in ether at −100 °C [257,258]:

TlClO_4_ + LiAlH_4_ → TlAlH_4_ + LiClO_4_. (80)

TlAlH_4_ decomposed at −80 °C (1.9 wt.% hydrogen content). Wiberg et al. tried the metathesis reaction between TiCl_3_ and LiAlH_4_ in ether at −115 °C [258]. However, no Tl^+3^-alanate could be isolated from the reaction of TlCl_3_ and LiAlH_4_, with the product spontaneously decomposing at −110 °C [258,259]. A marginal stabilization was achieved when a Cl^−^ substituted an [AlH_4_]^−^ ion: TlCl(AlH_4_)_2_ was produced by the reaction between TlCl_3_ and AlH_3_ in ether at −115 °C in the presence of AlH_3_·AlCl_3_ [259]. TlCl(AlH_4_)_2_ decomposed at −95 °C [259].

#### 3.5.4. Tin Alanate

Sn(AlH_4_)_4_ (6.6 wt.% hydrogen content) was produced by the reaction between SnCl_4_ and LiAlH_4_ in ether at −80 °C [260]:

SnCl_4_ + 4LiAlH_4_ → Sn(AlH_4_)_4_ + 4LiCl. (81)

Sn(AlH_4_)_4_ decompose at −40 °C. The decomposition products are Sn, Al and H_2_. 

### 3.6. Alanates of Lanthanides and Actinides

#### 3.6.1. Lanthanum, Cerium, Praseodymium and Neodymium Alanates

La, Ce, Pr, and Nd alanates were produced by metathesis that was assisted by mechanical milling of the corresponding trichlorides and NaAlH_4_ (in excess 1:3) under hydrogen pressure (1–15 bar) [261]. The expected products, M(AlH_4_)_3_, M = La, Ce, Pr, and Nd, are unstable and decompose during ball milling. Instead of M(AlH_4_)_3_, alumino-hydrides of stoichiometry MAl*_x_*H*_y_* were obtained (very close to MAlH_6_ stoichiometry). Thermolysis of the MAlH_6_ (M = Ce, Pr, and Nd) materials demonstrated two-steps of decomposition, except for LaAlH_6_ [261]. The first step is associated with the decomposition of the alanate. Meanwhile, the second step can be associated with the decomposition of the corresponding metal hydride and the formation of M-Al alloys. Although the decomposition pathway was proposed for Nd, based on the in-situ X-ray diffraction data that were presented by Weidenthaler et al., the reaction can be extrapolated for Ce and Pr [261]:

NdAlH_6_ → NdH_3_ + Al + 3/2H_2_ (82)

NdH_3_ +4Al → NdAl_4_ +3/2H_2_ (83)

**Figure 20:**
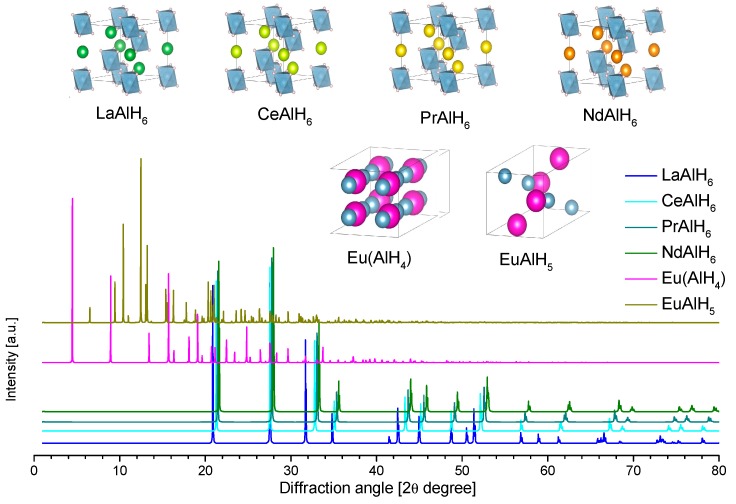
Crystal structure of lanthanides alanates and their calculated diffraction patterns (λ = Cu_kα1_).

Table 12 summarizes the hydrogen content, hydrogen released, decomposition temperatures, and crystal structure data [261]. The experimental X-ray diffraction patterns of MAl*_x_*H*_y_* were compared to the DFT calculations of hypothetical MAlH_6_ materials. Figure 20 presents the expected X-ray diffraction patterns and the structures. 

#### 3.6.2. Europium Alanate

Eu(AlH_4_)_2_ was produced by the metathesis reaction of EuCl_2_ + 2NaAlH_4_ or EuCl_3_ + 3NaAlH_4_. The reaction was performed by means of mechanical milling in a hydrogen atmosphere (1–15 bar) and different milling times (180 min seems enough time) [44]. Independently of the initial oxidation state of Eu ion, Eu^2+^, or Eu^3+^, the final alanate was Eu^2+^, i.e., Eu(AlH_4_)_2_. Additionally, NaEu_2_Cl_6_ was observed as an intermediary. Eu(AlH_4_)_2_ has a hydrogen content of 3.76 wt.%. Pommerin et al. demonstrated a hydrogen release of about 1.8 wt.% (including the NaCl load) in two steps [44]. The first step occurred at about 100–125 °C with the formation of EuAlH_5_. The second step occurred at about 200–225 °C. Further heating led to the formation of EuAl_4_. Rehydrogenation was achieved by milling at high hydrogen pressure (50, 200, or 300 bar). Unfortunately, the rehydrogenation was not achieved under 1000 bar of static H_2_ pressure; i.e., the temperature of rehydrogenation was not clearly indicated without milling. Further dehydrogenation demonstrated that the two-step reactions and temperature range are kept. However, a drastic reduction of the hydrogen release was found (about 0.8 wt.%) [44]. Partial crystallographic information was reported, i.e., no H position was determined (Table 13) [44]. Figure 20 presents the expected X-ray diffraction patterns and structures.

#### 3.6.3. Ytterbium Alanate

Yb(AlH_4_)_2_ was reported by Kost et al. in 1978 [239]. The compound has a hydrogen content of 3.43 wt.%. It was produced in cold ether by the metathesis reaction between LiH, Al, and a metal halide. Kost et al. reported that Yb(AlH_4_)_2_ is a yellow powder that decomposes at 70 °C. The decomposition products of Yb(AlH_4_)_2_ are the hydrides of Al and Yb [239]. The YbH_2_ is metastable at room temperature [262]. 

#### 3.6.4. Thorium-Aluminum Hydride

No records of thorium alanate were found; however, an intermetallic hydride of Th was found: Th_2_AlH_4_. The thorium-aluminum hydride can be easily obtained by the hydrogenation of the intermetallic Th_2_Al [263]. Th_2_Al needs activation at 450 °C in vacuum, followed by deuterium absorption at 0.15 bar and iced-water cooling [264]. The products were Th_2_AlD*_x_*, *x* = 3.9 ± 0.1, 2.7, and 2.3 [264]. Experimental and theoretical crystal structure of Th_2_AlH_4_ reasonably agreed on a *I4/mcm* space group with lattice parameters a = 7.626 Å, and c = 6.515 Å, and atomic positions Th (0.1656, 0.6656, 0), Al (0, 0, 0.25), and H (0.377, 0.8707, 0.1512) [265].

## 4. Cation-Mixed Alanates

Cation substitution has demonstrated utility in the tailoring of the thermodynamic and kinetic properties in borohydrides [22,266]. A similar approach has been applied to alanates, for which LiAlH_4_ or NaAlH_4_ are frequently used as starting materials due to their reactivity. These alanates react with other metal hydrides to form mixed cation alanates. The reactions can be generalized as [267]:

M’AlH_4_ + 2MH → M_2_M’AlH_6_, (84)

MAlH_4_ + MH + M´H → M_2_M´AlH_6_, M≠M´. (85)

Theoretical calculations had predicted the stability of alanates, such as LiNa_2_AlH_6_, K_2_LiAlH_6_, K_2_NaAlH_6_, K_2.5_Na_0.5_AlH_6_, LiMgAlH_6_, LiCaAlH_6_, NaCaAlH_6_, and KCaAlH_6_ [268,269]. Some of them have been successfully synthesized, as presented below.

### 4.1. Li-Na Alanates

Na_2_LiAlH_6_ can be obtained by the reaction of 2NaH and LiAlH_4_ in an organic solvent [270], in the solid-state at very high hydrogen pressure [270], by means of a mechanically activated reaction between NaH, LiH, and NaAlH_4_ (Equation (86)) [271], or 2NaH + LiAlH_4_ [272,273,274], or NaH + LiAlH_4_ [275], or 2NaAlH_4_ + LiH [276], or by the reactive mechanical milling of 2NaH+LiH+Al that was catalyzed with TiF_3_ under 30 bar of hydrogen pressure [277]. Wang et al. produced Na_2_LiAlH_6_ by the reaction between 2NaH and LiAH_4_ [274]. However, detailed study of the synthesis reaction pathway by X-ray diffraction demonstrated that, during mechanical milling, a metathesis reaction occurred to produce a mixture of LiH, NaAlH_4_, and residual NaH, i.e., the same reactants of Equation (86). 

NaH + LiH + NaAlH_4_ → Na_2_LiAlH_6_. (86)

LiNa_2_AlH_6_ was also observed during the electrochemical decomposition of NaAlH_6_ in the presence of Li [278]:

NaAlH_4_ + 3/2 Li → ½ Na_2_LiAlH_6_ + ½ Al + LiH. (87)

Na_2_LiAlH_6_ has a total hydrogen content of 7.03 wt.% and a theoretical reversible hydrogen storage of 3.51 wt.% (Equation (88)). Na_2_LiAlH_6_ has demonstrated reversibility (Equation (88)) [274,277], which is enhanced by the use of a catalysts, such as TiF_3_, TiFe_3_, TiCl_3_, CeO_2_, ZrCl_4_, TiBr_4_, CrCl_3_, AlCl_3_, TiO_2_, Y_2_O_3_, or MnCl_2_ [276,277,279,280].

Na_2_LiAlH_6_ ↔ 2NaH + LiH + Al + 3/2 H_2_. (88)

Dehydrogenation reaction (Equation (88)) without additives occurs between 190–250 °C and it releases about 3.35 wt.%. Further reactions involve NaH decomposition at 320–380 °C and finally the reaction of LiH with Al at 380–480 °C, with the formation of LiAl and H_2_. Wang et al. demonstrated a release of 6.73 wt.% and a re-hydrogenation level of 6.6 wt.% when heating up to 530 °C under vacuum, and 285 °C and 135 bar, respectively. Small amounts of Na_3_AlH_6_ have been observed during the dehydrogenation of Na_2_LiAlH_6_ [274,281]. Additives, such as TiF_3_, resulted in a low-temperature beginning of Na_2_LiAlH_6_ decomposition (~50 °C) [276,277,280]. Additionally, Al_3_Ti was found after dehydrogenation when Na_2_LiAlH_6_ is mixed with TiF_3_ [279,280]. 

**Figure 21:**
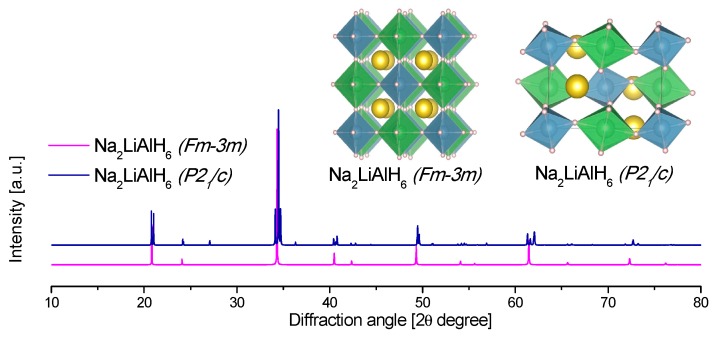
Crystal structure of Na-Li alanate and its calculated diffraction patterns (λ = Cu_kα1_).

First principle studies (before experimentation, i.e., synthesis and crystal structure determination) indicated that Na_2_LiAlH_6_ would have *P 2_1_/n* [282] or *P 2_1_/c* [283] symmetry, which is very close to *Fm-3m* symmetry [283]. Brinks et al. determined the group symmetry of Na_2_LiAlD_6_ as *Fm-3m*. This material consists of corning-sharing (AlD_6_) and (LiD_6_) octahedra, where each octahedron is surrounded by six octahedra (Table 14 and Figure 21) [284]. The deuterated Na_2_LiAlD_6_ was produced by the ball milling of NaAlD_4_ and LiAlD_4_ [284]. 

**Figure 22:**
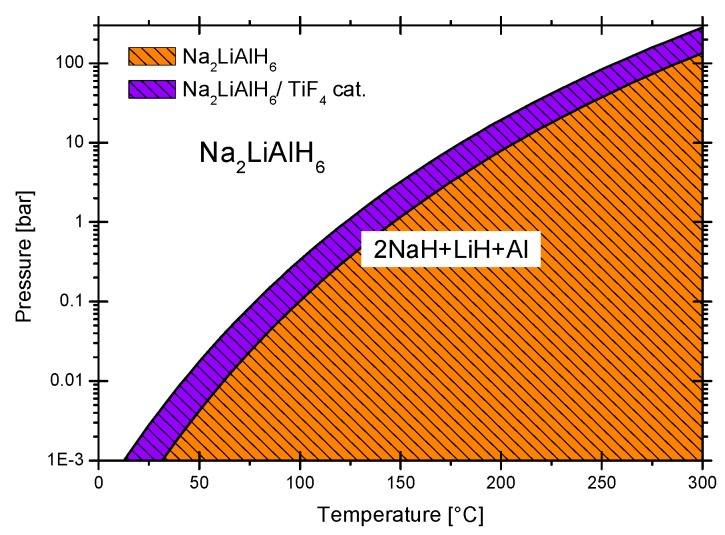
Phase diagram of 2NaH + LiH + Al vs. Na_2_LiAlH_6_. Data adapted from references [274,285], $\ln\left(p\right)=-\frac{7685.3}{T}+18.3$ for un-catalyzed material, and $\ln\left(p\right)=-\frac{6894.9}{T}+17.0$ for material catalyzed with TiF_4_. In the original formulae, *p* is in atm, and *T* in Kelvin.

The research group of Prof. Q. Wang performed a complete study regarding the determination of the (*p*, T) equilibrium of reaction (88) with and without TiF_4_ as a catalyst (Figure 22) [274,285]. The results indicate that the catalyst moves to higher pressure the equilibrium towards Na_2_LiAlH_6_ formation at a given temperature; or conversely a reduction of the equilibrium temperature at a given pressure. Fonneløp et al. revealed that the addition of 10 mol% of TiF_3_ to Na_2_LiAlH_6_ induced hydrogen release at temperatures as low as 50 °C [281]. In such a case, the dehydrogenation pathway changes from a one-step process (Equation (88)) to a two-step process, with the formation of Na_3_AlH_6_ as the intermediary. Between 50–180 °C, the decomposition reaction was described as:

Na_2_LiAlH_6_ → 2/3 Na_3_AlH_6_ + LiH + 1/3 Al + 1/2 H_2_. (89)

Further heating (180–225 °C) leads to the usual decomposition reaction of Na_3_AlH_6_. 

Finally, the other possible combination of Li, Na, Al, and H would be as Li_2_NaAlH_6_. However, attempts to synthesize this material have been unsuccessful. The attempts involve the synthesis in organic solvents, such as Me_2_O (160 °C, 12 h), or by ball-milling [267]. As proposed by Santhanam et al. [169], Li_2_NaAlH_6_ is not formed at all under the tested conditions, or it disproportionates Na_2_LiAlH_6_, LiH and LiAlH_4_. 

### 4.2. Li-K Alanates

**Figure 23:**
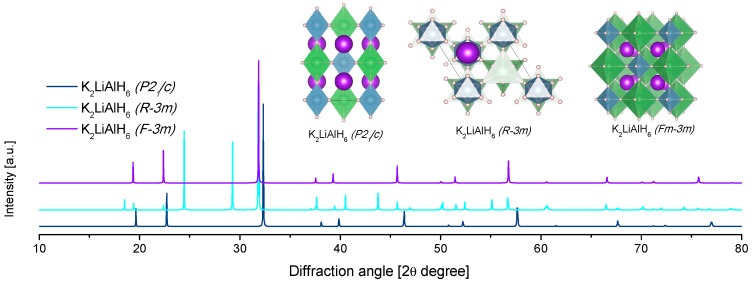
Crystal structure of Li-K alanate and its calculated diffraction patterns (λ = Cu_kα1_).

K_2_LiAlH_6_ was reported in 2005 by Graetz et al. [267]. K_2_LiAlH_6_ was produced by the ball-milling of 2KH + LiAlH_4_ [267]. Graetz et al. determined an *Fm-3m* structure for K_2_LiAlH_6_. However, in their paper, they recognized that the diffraction pattern was not suitable for Rietveld analysis [267]. Briefly, after that, Rönnebro et al. performed the mechanical milling of the same precursors followed by a heating treatment of the pelletized sample at 320–330 °C and 700 bar for 1–2 days. By doing this, K_2_LiAlH_6_ was crystallized, and its crystal structure was determined to have *R3m* symmetry (Table 15) [286]. As in the case of Na_2_LiAlH_6_, theoretical calculations (predating synthesis and crystal structure determination) predicted that K_2_LiAlH_6_ would have *P 2_1_/n* symmetry (Table 15 and Figure 23) [282,283]. The differences between the calculated and the experimental data could be related to the temperature of calculation (0 K) versus the temperature of synthesis and testing (near room temperature). 

K_2_LiAlH_6_ has a total hydrogen content of 5.11 wt.% and a possible reversible hydrogen storage of 2.56 wt.%. The dehydrogenation of K_2_LiAlH_6_ was performed at 227 °C, while rehydrogenation was performed at 300 °C and up to 10 bar [267]. The rehydrogenation achieved 2.3 wt.% hydrogen storage, i.e., approximately 90% of the theoretical value. However, the reaction time was very long, around 280 h; and, perhaps a higher hydrogenation pressure would improve kinetics. 

Regarding other Li-K alanates and similar to the Li_2_NaAlH_6_ case, no Li_2_KAlH_6_ has been produced so far [169].

### 4.3. Li-Mg Alanates

The mixed alanate LiMg(AlH_4_)_3_ has a hydrogen content of 9.7 wt.%; LiMg(AlH_4_)_3_ is known since 1979 by the work of Bulychev et al. [287]. It can be produced by the metathesis reaction between LiAlH_4_ and MgCl_2_ [165,220,288]:

3LiAlH_4_ + MgCl_2_ → LiMg(AlH_4_)_3_ + 2LiCl. (90)

Reaction (90) can be performed in an organic solvent or assisted by mechanical milling. The decomposition of LiMg(AlH_4_)_3_ is a two-step process [289,290]:

LiMg(AlH_4_)_3_ → LiMgAlH_6_ + 2Al + 3H_2_ (100–130 °C) (91)

LiMgAlH_6_ → LiH + MgH_2_ + Al + 3/2 H_2_ (150–180 °C) (92)

**Figure 24:**
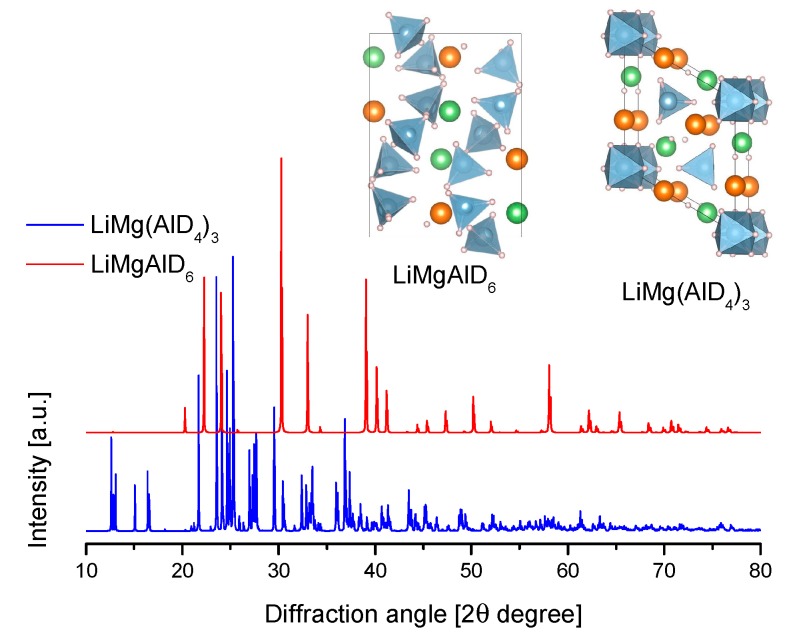
Crystal structure of Li-Mg alanates and its calculated diffraction patterns (λ = Cu_kα1_)

The addition of graphitic nanofibers can reduce the dehydrogenation temperatures [291]. Addition of TiF_3_ leads to the decomposition of the mixed alanate even during ball-milling [290]. Attempts of re-hydrogenation were unsuccessful, even at high pressures [289,290]. The structure of LiMg(AlH_4_)_3_ consists of a corner-sharing network of alternating [AlH_4_]^−^ tetrahedra and (LiH_6_) and (MgH_6_) octahedra (Table 16 and Figure 24) [288]. The structure of LiMgAlH_6_ consists of alternating AlMg_3_ and Al_2_Li_3_ layers; in the Al_2_Li_3_ layer, the [AlH_6_]^−^ octahedra share edges with three (LiD_6_) octahedra [206,290]. 

### 4.4. Li-Ca Alanates

LiCa(AlH_4_)_3_ has a total hydrogen content of 8.6 wt.%; thus, it appears as a very attractive hydrogen storage material. LiCa(AlH_4_)_3_ was produced by the metathesis reaction between LiAlH_4_ and CaCl_2_, utilizing mechanical milling [292]:

3LiAlH_4_ + CaCl_2_ → LiCa(AlH_4_)_3_ + 2LiCl. (93)

LiCa(AlH_4_)_3_ (plus LiCl) starts decomposing at 120 °C and it ends at about 180 °C. Liu et al. proposed the formation of LiCaAlH_6_ in the first dehydrogenation step [292]. In the second step (180–300 °C), LiCaAlH_6_ decomposed to form Al, CaH_2_, and LiH. The two steps released 6 wt.% of hydrogen [292]:

LiCa(AlH_4_)_3_ → LiCaAlH_6_ + 2Al + 3H_2_ (94)

LiCaAlH_6_ → CaH_2_ + LiH + Al + 3/2 H_2_ (95)

**Figure 25:**
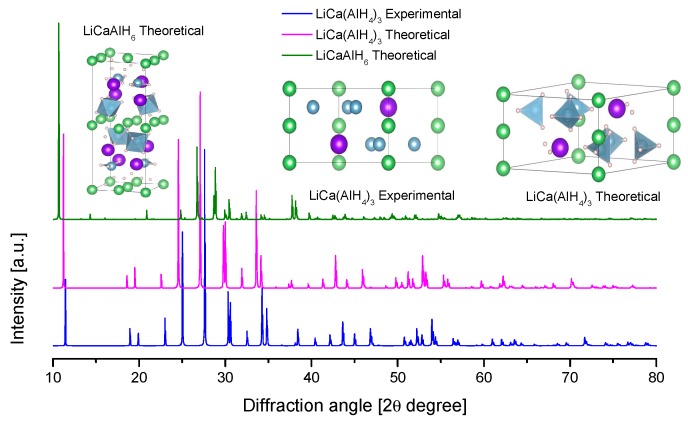
Crystal structure of Li-Ca mixted alanate and its calculated diffraction patterns (λ = Cu_kα1_).

In the second step, some CaH_2−*x*_Cl*_x_* was detected. No information regarding possible re-hydrogenation was found. The crystal structure of LiCa(AlH_4_)_3_ was experimentally determined as the space group *P6_3_/m* (Table 17 and Figure 25) [292]. Theoretical research confirmed this symmetry and contributed to determining the hydrogen atomic positions (Table 17) [293]. The complete crystal structure of LiCaAlH_6_ was predicted from the theoretical calculations [294]. 

### 4.5. Na-K Alanates

K_2_NaAlH_6_ is the only reported mixed Na-K alanate. This material has a total hydrogen content of 4.46 wt.%. K_2_NaAlH_6_ can be produced by the reaction assisted by ball-milling between KH and NaAlH_4_ in a 2:1 molar relation, with or without hydrogen pressure (10 bar) [295,296]. K_2_NaAlH_6_ decomposes into simple hydrides, Al and hydrogen gas at ~352 °C [296,297]:

K_2_NaAlH_6_ → 2KH + NaH + Al + 3/2 H_2_ (96)

**Figure 26:**
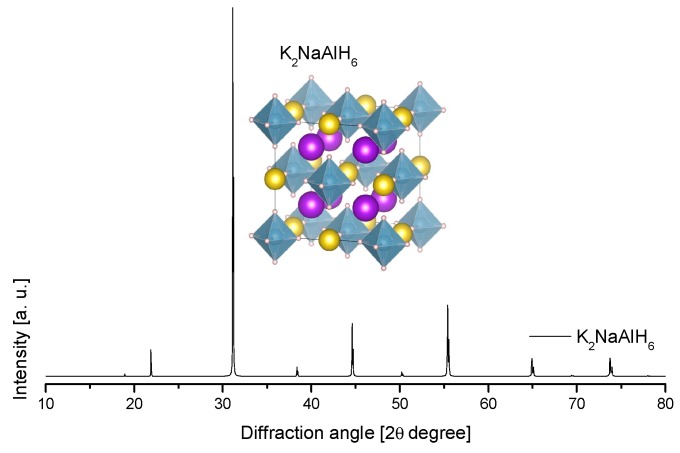
Crystal structure of Na-K mixed alanate and its calculated diffraction pattern (λ = Cu_kα1_).

The addition of TiCl_3_, TiF_3_, graphene, or carbon nanotubes slightly reduced the dehydrogenation temperature, with TiF_3_ being the most effective material [296]. K_2_NaAlH_6_ is reported to store hydrogen reversible; however, full capacity was not recovered [295]. K_2_NaAlH_6_ is reported as a cubic close-packed structure of isolated [AlH_6_]^3−^ octahedra; the octahedral interstices are occupied by Na^+^ ions, while the tetrahedral interstices are filled with K^+^ ions (Table 18, Figure 26) [295].

## 5. Anion Substitution

Ion size and oxidation state make, in principle, F^−^ ions suitable for substituting H^−^ ions in some hydrogen storage compounds, such as hydrides [298], borohydrides, or alanates [299]. The substitution could tune the thermodynamics, with the goal being to reduce the dehydrogenation temperature [299]. Perhaps the clearest example of this is the production of Na_3_AlH_6−*x*_F*_x_* from NaF and Al [300]. However, despite reducing the enthalpy of the first dehydrogenation, the reversibility of the system was compromised [300]. Other examples of anion substitution, despite being less studied, included K_3_AlH_6−*x*_F*_x_* [301] and CaAlF*_x_*H_5−*x*_ [219]. Unfortunately, limited information regarding these systems can be found, thus experimental and/or theoretical studies should be performed in the future. 

## 6. Techniques of Characterization of Alanates

The most common physicochemical characterization techniques for hydrogen storage materials, and thus alanates, are X-ray diffraction (in-situ, ex-situ, with synchrotron or conventional X-ray sources), and spectroscopies, such as Infrared and Raman. Other vibrational spectroscopy techniques, such as Inelastic Neutron Scattering (INS), Nuclear Resonant Inelastic X-ray Scattering Spectroscopy (NRIXS), or Photoacoustic (PA) Infrared Spectroscopy are far less widespread. The main results of X-ray diffraction studies were presented along with the description of each alanate. Thus, we did not include a special section for it. On the other hand, the characterization of alanates by IR and Raman Spectroscopies is also frequently used due to the relatively low cost of equipment and the relative simplicity of sample preparation for such tests. Therefore, we present IR and Raman spectroscopies in this review. 

### Fourier Transformed Infrared Spectroscopy (IR) and Raman Spectroscopy

**Figure 27:**
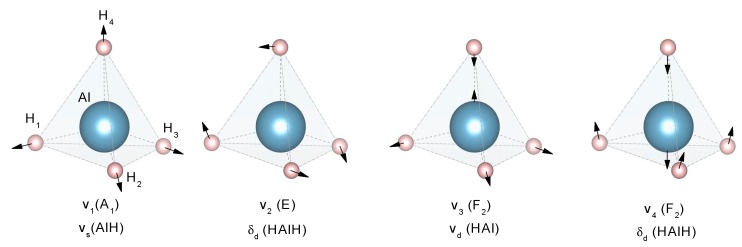
Normal modes of vibration of tetrahedral [AlH_4_]^−^. Adapted from reference [302].

**Figure 28:**
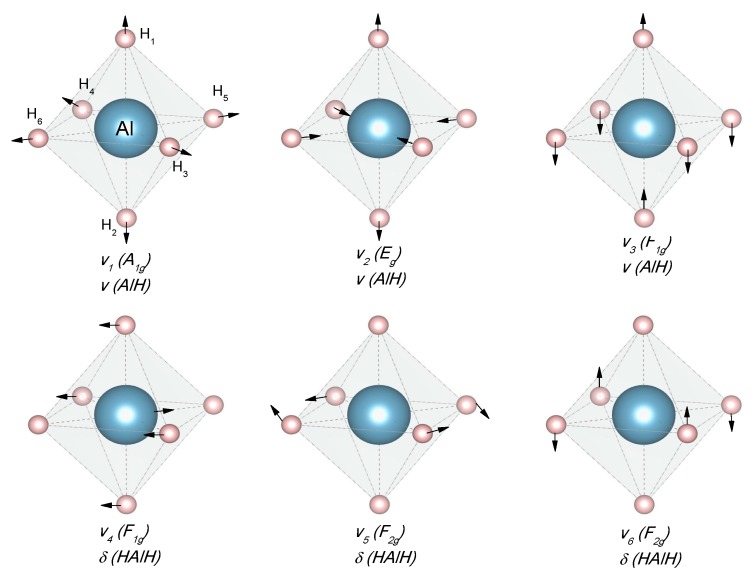
Normal modes of vibration of octahedral [AlH_6_]^3−^. Adapted from reference [302].

Vibrational transitions can be observed as infrared or Raman spectra. Although frequently, these two techniques are complementary, their physical origins are different [302]. IR absorption spectra originate from photons in the infrared region that are absorbed by transitions between two vibrational levels of the molecule in the electronic ground state. Raman spectra have their origin in the electronic polarization that is caused by ultraviolet, visible, and near-IR light [302]. The observed vibration modes depend on factors, such as the molecular symmetry, identity of atoms, and bond energies, i.e., the kinetic and potential energies of the system. The kinetic energy is determined by the masses of the individual atoms and their geometrical arrangement in the molecule. On the other hand, the potential energy arises from the interaction between the individual atoms and it is described in terms of the force constants [302]. For the alanates, the common structures are the tetrahedral [AlH_4_]^−^ and octahedral [AlH_6_]^3−^ units. Figure 27 illustrates the four normal modes of vibration of a tetrahedral [AlH_4_]^−^. All four vibrations are Raman-active, whereas only ν_3_ and ν_4_ are infrared active [302]. Octahedral molecules have six normal modes of vibration; of these, vibrations ν_1_, ν_2_, and ν_5_ are Raman-active, whereas only ν_3_ and ν_4_ are infrared-active (Figure 28) [302]. 

**Figure 29:**
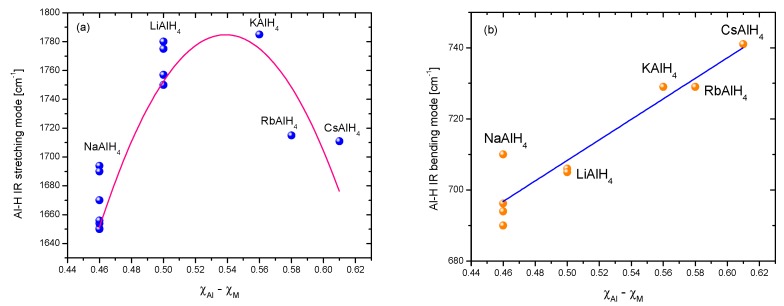
Most intense peak of infrared vibrations in the group 1 alanates, MAlH_4_. (**a**) Stretching, (**b**) Bending.

The vibrational spectra of alanates are frequently classified as external and internal. The external vibrations are due to the vibration of the whole crystal structure. Meanwhile, the internal vibrations are due to the [AlH_4_]^−^ ion, which has four active vibrational modes in Raman and only two in infrared [303]. Some of these features are shared with other materials of similar structure, for example, the borohydrides [304]. The infrared active modes of the [AlH_4_]^−^ ion are the asymmetric stretching modes in the region 1600–2000 cm^−1^ and the bending modes in the region 700–900 cm^−1^ [305]. Some representative data are collected in Table 19 and Table 20. As a generally accepted trend of infrared vibrations in the alanates of group 1, the stretching modes, in wavenumbers, roughly decrease with increasing mass of the cation [306]. Meanwhile, the bending modes are unaffected by the counter-ion [305,306]. Other correlations between the stretching and bending peaks (or regions) versus ionization energy, electronegativities, or bond distance have been proposed [302]. Indeed, we tried to find correlations with these parameters. However, we obtained the best results by using the difference in the electronegativities between Al and the counter-cation or the counter-cation ion size. In Figure 29 we present a correlation between the most intense stretching and bending IR peak of MAlH_4_ (M = group 1 metals, [AlH_4_]^−^ tetrahedra) versus the difference in electronegativities of Al and the metal. The electronegativity scale was the Allred–Rochow [307]. The IR data that were obtained by Adicks et al. in pure crystalline materials [177] were complemented by data published in several experimental and theoretical reports compiled in this review [167,308,309,310,311,312,313,314,315,316,317,318,319,320]. The data reflects the significant dispersion of results. The NaAlH_4_ data are the most common and particularly disperse, which is probably due to the diversity in the material history, such as milling, doping, or cycling [318]. The quantity of available IR data on K, Rb, and Cs- alanates is rather scarce. Still, some tendencies were found; there is a bell-shape dispersion of the Al-H stretching frequency (most intense peak) versus the difference of electronegativity between Al and the group 1 metal. Meanwhile, there is an almost linear increase of the Al-H bending frequency (most intense peak). This can be related to the changes in the geometry of the alanates, along with the group.

**Figure 30:**
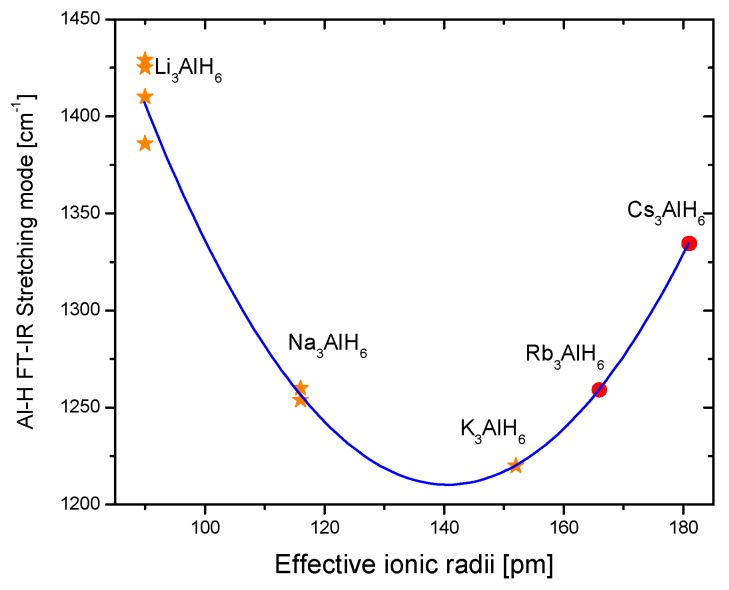
Most intense peak of infrared vibrations in the group 1 intermediaries, M_3_AlH_6_. The red dots are an extrapolation based on the fitted curve.

The octahedral ion [AlH_6_]^3−^ that is present in the so-called intermediaries of alanates also shows infrared and Raman active modes. From the 15 normal vibration modes of a group with octahedral symmetry, two modes are active in the infrared, and three modes are active in the Raman [321]. In Figure 30, we present a correlation between the stretching IR most intense peak of M_3_AlH_6_ (M = group 1, [AlH_6_]^3−^ octahedra) versus the effective ionic radii [307]. The available data for the so-called intermediaries of alanates of group 1 (M_3_AlH_6_) are scarcer than for the tetrahedral alanates, i.e., MAlH_4_. Thus, the correlation was constructed with data of Li, Na, and K [167,307,318,322,323,324,325,326,327]. The red dots of Rb_3_AlH_6_ and Cs_3_AlH_6_ are an extrapolation based on the fitted curve. 

**Figure 31:**
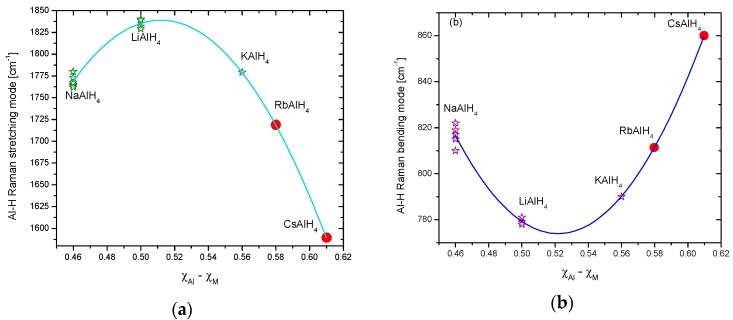
Most intense Raman peak in the group 1 alanates. (**a**) Stretching and (**b**) Bending modes. The red dots are an extrapolation based on the fitted curve.

In Figure 31, we present a correlation between the stretching and bending Raman most intense peak versus the difference in electronegativities between Al and the metal of MAlH_4_ (M = group 1). In general, there are less Raman data available than IR data. In both stretching and bending Raman modes, the correlation with the difference in electronegativity is not linear. The reported data were found only for Li, Na, and K-alanates. Thus, the Rb and Cs-alanates data are an extrapolation, pending future reports to corroborate this forecast. 

Not enough IR or Raman data are available for group 2 (apart from Mg and Ca) and the rest of alanates of the periodic table. Additionally to the Figure 29, Figure 30 and Figure 31, an attempt to find trends that include the double-metal alanates of groups 1 and 2 was performed; no clear trends were found. This can open the possibility of theoretical and experimental studies to obtain these missing data and to obtain general rules that correlate structure and spectroscopic properties.

## 7. Thermodynamics

A dehydrogenation enthalpy of about 40 kJ/mol is required in order to meet the dehydrogenation temperature compatible with PEMFCs [328]. This enthalpy value roughly means an equilibrium pressure of 1 bar at room temperature. The equilibrium pressure is a function of the temperature, the dehydrogenation enthalpy, and entropy. It is described by the Van’t Hoff equation [328]:

(97) $$\ln\left(\frac{p_{eq}}{p_{eq}^{0}}\right)=\frac{\Delta H}{R}\times \frac{1}{T}-\frac{\Delta S}{R}$$

Δ*S* mostly corresponds to the change from molecular hydrogen gas to dissolved solid hydrogen [328]. It amounts approximately to the standard entropy of hydrogen (130 J·K^−1^mol^−1^) and is, therefore, frequently taken as a constant for all metal-hydrogen systems [328]. Δ*H* must be the dehydrogenation reaction enthalpy and each material must report it. However, the formation enthalpy is sometimes used instead, particularly if the material is a metal and its hydride. The enthalpies of formation and dehydrogenation have been related, directly or indirectly, to the bond energy, i.e., the stability of the compound [328,329]. The reported dehydrogenation enthalpies were used to construct the phase diagrams that are presented in this review. The representative values are condensed in Table 21, altogether with formation enthalpies and the activation energies. The thermodynamic data is concentrated mainly in the alanates of group 1, a lot of data is missing on other alanates. The calculated and experimental data of formation enthalpy and dehydrogenation enthalpy show good correlation. However, an in-deep comment on the dispersion of the thermodynamic data is needed. Along with the several consulted papers, different experimental techniques and conditions were used to determine the thermodynamic data. The most used techniques are the differential scanning calorimetry (with variations, such as high-pressure, with or without hydrogen flow, different of values of flows, etc.), pressure-composition isotherms, and theoretical calculations (different levels of theory, programs, basis sets, etc.). Thus, the natural result is the dispersion of data. Perhaps, a standard method will be advisable. Meanwhile, the activation energies present the most disperse values, which is due to the additive and the history of the materials (mechanical milling, purification, recrystallization, cycling, etc.). Additionally, some of the original data are explicitly related to the released mol of H_2_, meanwhile, other data is not clearly reported of mol of which compound is related. 

## 8. Conclusions and Perspectives

NaAlH_4_ and KAlH_4_ stand out among all of the alanates due to their acceptable hydrogen content and reversibility. Perhaps for light-duty vehicles applications, an option will be the NaAlH_4_, where the catalyst performance is essential. In that subject, along with the consulted papers, the Ti-based catalyst could be limited in the long-term because of the progressive change in the oxidation state of Ti, associated with the decay of performance. Perhaps, lanthanide-metals compounds could be the solution. However, more research on extensive cycling must be done: There is not enough data up to now on the long-term performance of Ce-catalysts on NaAlH_4_. On the other hand, KAlH_4_ can be suitable for niche applications where the high-temperature dehydrogenation is not an issue. However, there is no data regarding extensive cycling.

During the preparation of this review, the compilation of alanates beyond the group 1 and 2 was a good surprise. Many of them have a reasonable good dehydrogenation temperature and hydrogen content. Others can be viewed just as a chemical curiosity. In general, the reports of the alanates of transition metals and main group are very old. Perhaps, re-visiting and updating the information of these alanates with new synthesis and characterization techniques could provide new approaches for solving the hydrogen storage problem. 

Despite that the formation of reactive composite materials has proven useful in other hydrogen storage materials, this approach seems not so useful in the alanate family. However, the formation of double cation alanates seems to be attractive for improving the dehydrogenation temperature without the sacrifice of the hydrogen content. The anion substitution is explored to a limited extent in the alanates family, and this modification should be studied deeply. 

## Figures and Tables

**Table 1:** Crystallographic data of alanes.

Compound	Space Group, Cell Dimensions [Å] and Angles [°]	Atomic Coordinates
α-AlD_3_	*(R-3c)* No. 167 [66] a = 4.227; b = 4.227; c = 11.244 α = β = 90; γ = 120	Al: 0, 0, 0 D: 0.63, 0, ¼
α’-AlD_3_	*(Cmcm)* No. 63 [47] a = 6.470(3); b = 11.117(5); c = 6.562(2) α = β = 90; γ = 90	Al1: 0, ½, 0 Al2: ¼, ¼, 0 D1: 0, 0.197(2), 0.451(4) D2: 0.312(2), 0.1000(14), 0.047(3) D3: 0, 0.465(3), ¼ D4: 0.298(4), 0.277(2), ¼
β-AlD_3_	*(Fd-3m)* No. 227 [67] a = 9.0037(1); b = 9.0037(1); c = 9.0037(1) α = β = γ = 90	Al1: ½, 0, 0 D1: 0.4301(1); 0.125; 0.125
γ-AlD_3_	*(Pnnm)* No. 58 [68] a = 7.3360(3); b = 5.3672(2); c = 5.7562(1) α = β = γ = 90	Al1: 0, 0, 0 Al2: 0.4174(5), 0.7127(6), 0 D1: 0.2044(9), 0.8269(11), 0 D2: 0.3668(10), 0.3931(13), 0 D3: 0, ½, ½ D4: 0.4174(6), 0.7038(8), 0.3009(6)

**Table 2:** Crystallographic data of Li-alanates.

Compound	Space Group, Cell Dimensions [Å] and Angles [°]	Atomic Coordinates
α-LiAlD_4_	(P21/c) No. 14 [78] a = 4.8254(1); b = 7.8040(1); c = 7.8968(1) α = 90; β = 112.268(1); γ = 90	Al: 0.1428(2), 0.2013(1), 0.9311(1) Li: 0.5601(12), 0.4657(6), 0.8236(6) D1: 0.1902(10), 0.0933(8), 0.7710(6) D2: 0.3526(10), 0.3726(7), 0.9769(6) D3: 0.2384(11), 0.0840(7), 0.1141(7) D4: 0.8024(14), 0.2644(7), 0.8689(8)
β-LiAlH_4_	(I 41/a) No. 88 [76] a = 4.6611; b = 4.6611; c = 10.5219 α = β = γ = 90	Li: 0, ¼, 0.625 Al: 0, ¼, 0.125 H: 0.2527, 0.4237, 0.5413
γ-LiAlH4	(Pnma) No. 62 [76] a = 6.4667; b = 5.3478; c = 6.5931 α = β = γ = 90	Li: 0.2428, ¼, 0.2467 Al: 0.513, ¼, 0.8221 H1: 0.3067, ¼, 0.9617 H2: 0.7162, ¼, 0.9631 H3:0.4889, 0.9833, 0.2943
Li_3_AlH_6_	(R-3) No. 148 [81] a = 8.0389(2); b = 8.0389(2); c = 9.4755(5) α = β = 90, γ = 120	Al1: 0, 0, 0 Al2: 0, 0, ½, Li: 0.966(2), 0.236(3), 0.3007(17) D1: 0.8325(11), 0.8030(7), 0.1008(6) D2: 0.1593(10), 0.1799(8), 0.3884(6)

**Table 3:** Crystallographic data of Na-alanates.

Compound	Space Group, Cell Dimensions [Å] and Angles [°]	Atomic Coordinates
NaAlH_4_	(*I 4_1_/a*) No. 88 [118] a = b = 5.020(2); c = 11.330(3) α = β = γ = 90	Al: 0, 0, 0 Na: 0, 0, ½ H: 0.228(1) 0.117(2) 0.838(9)
Na_3_AlH_6_	(*P 2_1_/c*) No. 14 [121] a = 5.4145(3); b = 5.5402(3); c = 7.7620(4) α = 90; β = 89.871(4), γ = 90	Al: 0, 0, 0 Na1: 0, 0, ½ Na2: −0.00129(5), 0.46129(4), 0.25008(4) H1: 0.0918, 0.0352, 0.2207 H2: 0.222, 0.3283, 0.5454 H3:0.1649, 0.2689, 0.95
Na_3_AlD_6_	(*P 2_1_/c*) No. 14 [122] a = 5.390(2); b = 5.514(2); c = 7.725(3) α = 90; β = 89.86(3), γ = 90	Na1: 0, 0, ½ Na2: −0.006(5), 0.461(4), 0.252(5) Al: 0, 0, 0 D1:0.091(3), 0.041(3), 0.215(3) D2: 0.234(3), 0.328(3), 0.544(3) D3: 0.165(3), 0.266(3), 0.944(3)

**Table 4:** Crystallographic data of K-alanates.

Compound	Space Group, Cell Dimensions [Å] and Angles [°]	Atomic Coordinates
KAlD_4_	(*Pnma*) No. 62 [173] a = 8.8514(14); b = 5.8119(8); c = 7.3457(11) α = β = γ = 90	K: 0.1839(12), ¼, 0.1522(17) Al: 0.5578(11), ¼, 0.8209(13) D1: 0.4018(10), ¼, 0.9156(9) D2: 0.7050(9), ¼, 0.9630(12) D3: 0.4209(6), 0.9741(8), 0.3098(7)
α-K_3_AlH_6_	(*P 2_1_/c*) No. 14 [175] a = 6.1771; b = 5.8881; c = 8.6431 α = 90; β = 89.3, γ = 90	K1: 0, 0, ½ K2: −0.0058, 0.4828, 0.2544 Al: 0, 0, 0 H1: 0.0617, 0.0089, 0.2042 H2: 0.2799, 0.3136, 0.5349 H3: 0.1786, 0.2281, 0.9652
β-K_3_AlH_6_	(*I 4/mmm*) No. 139 [175] a = b = 4.4441; c = 7.8098 α = β = γ = 90	K1: 0, 0, ½ K2: 0, ½, ¼ Al1: 0, 0, 0 H1: 0, 0, 0.2128 H2: 0.3429, 0, 0
γ-K_3_AlH_6_	(*Pnnm*) No. 58 [175] a = 10.8885; b = 10.2576; c = 2.5538 α = β = γ = 90	K1: 0.2347, 0.03444, 0 K2: 0.55047, ¾, 0 K3: 0.691, 0.2178, 0 Al1: ½, ½, 0 Al2: 0, ½, 0 H1: 0.9388, 0.0715, 0 H2: 0.5928, 0.3931, 0 H3: 0.3085, 0.3814, 0 H4: 0.0632, 0.3708, 0 H5: 0.4194, 0.0352, 0 H6: 0.8387, 0.3512, 0

**Table 5:** Crystallographic data of Rb-alanates.

Compound	Space Group, Cell Dimensions [Å] and Angles [°]	Atomic Coordinates
α-RbAlD_4_	(*Pnma*) No. 62 [176] a = 9.2862(6); b = 5.9392(3); c = 7.5784(6) α = β = γ = 90	Rb: 0.1813(4), ¼, 0.1574(7) Al: 0.5639(6), ¼, 0.8121(7) D1: 0.4045(7), ¼, 0.9073(7) D2: 0.6884(7), ¼, 0.9615(8) D3: 0.4204(4), 0.9691(6), 0.3080(6)
α-RbAlD_4_	(*Cmc2_1_*) No. 36 [176] a = 3.9933; b = 14.6472; c = 6.4933 α = β = γ = 90	Rb: ½, 0.6206, 0.2833 Al: ½, 0.1154, 0.7607 D1: ½, 0.7996, 0.0670 D2: ½, 0.1717, 0.9990 D3: ½, 0.5992, 0.7814 D4: ½, 0.9888, 0.1074

**Table 6:** Crystallographic data of Cs-alanates.

Compound	Space Group, Cell Dimensions [Å] and Angles [°]	Atomic Coordinates
CsAlD_4_ (o)	(*Pnma*) No. 62 [185] a = 9.8847(5); b = 6.15949(29); c = 7.9182(4) α = β = γ = 90	Al: 0.55462(33), ¼, 0.80887(30) D1: 0.5755(4), 0.04042(31), 0.69536(32) D2: 0.6641(6), ¼, 0.9620(5) D3: 0.4017(5), ¼, 0.8868(6) Cs: 0.1847(4), ¼, 0.1652(8)
CsAlD_4_ (t)	(*I 4_1_/a*) No. 88 [185] a = b = 5.67231(9); c = 14.2823(5) α = β = γ = 90	Al: 0, ¾, 0.875 Cs: ½, ¾, 0.125 D1: 0.19658(31), 0.7115(9), 0.95567(12) D2:0.25993(26), 0.7644(19), 0.92159(17)

**Table 7:** Calculated crystallographic data of Be-alanates.

Compound	Space Group, Cell Dimensions [Å] and Angles [°]	Atomic Coordinates
α-BeAlH_5_	(*P2_1_*) No. 4 [192] a = 4.790; b = 4.324; c = 6.227 α = γ = 90; β = 89.408	Be: 0.002, 0.230, 0.623 Al: 0.243, 0.990, 1.000 H1: 0.247, 0.162, 0.749 H2: 0.001, 0.740, 0.902 H3: 0.501, 0.740, 0.914 H4: 0.240, 0.821, 0.251 H5: 0.890, 0.965, 0.515
β-BeAlH_5_	(*C2/c*) No. 15 [192] a = 5.959; b = 7.008; c = 6.241 α = γ = 90; β = 116.205	Be: 0, 0.333, 0.250 Al: 0,0,0 H1: 0, 0.904, 0.250 H2: 0.902, 0.777, 0.881 H3: 0.688, 0.044; 0.913

**Table 8:** Crystallographic data of magnesium alanates.

Compound	Space Group, Cell Dimensions [Å] and Angles [°]	Atomic Coordinates
Mg(AlH_4_)_2_	*(P −3 m 1)* No. 164 [202] a = b = 5.1949(2); c = 5.8537(2) α = 90; β = 90; γ = 120	Mg: 0, 0, 0 Al: 0.3333, 0.6667, 0.7057(5) H1: 0.3333, 0.6667, 0.439(2) H2: 0.1589(14), −0.1589(14), 0.804(2)
MgAlH_5_	*(P 2_1_ 2_1_ 2_1_)* No. 19 [206] a = 4.55; b = 4.26; c = 13.024 α = 90; β = 90; γ = 90	Mg: −0.2504, −0.2466, −0.3204 Al: 0.2486, 0.2528, −0.4083 H1: −0.4756, −0.0559, 0.4069 H2: −0.03, 0.0912, 0.3051 H3: 0.4719, −0.0516, −0.4063 H4: 0.0284, 0.0975, −0.3045 H5: −0.0024, 0.0916, −0.4994
α-MgAlH_5_	*(P* 21 /*c)* No. 14 [207] *a* = 4.7499; *b* = 8.8127; *c* = 6.6281 α = 90; β = 90; γ = 109.75	Mg: 0.527, 0.985, 0.253 Al: 0.092, 0.245, 0.395 H1: 0.400, 0.121, 0.444 H2: 0.349, 0.390, 0.495 H3: 0.121, 0.592, 0.201 H4: 0.197, 0.862, 0.142 H5: 0.130, 0.305, 0.156
β-MgAlH_5_	*(Cc)* No. 9 [207] *a* = 7.8033; *b* = 5.7251; *c* = 6.7393 α = 90; β = 90; γ = 115.39	Mg: 0.542, 0.025, 0.257 Al: 0.000, 0.000, 0.000 H1: 0.008, 0.924, 0.256 H2: 0.201, 0.289, 0.034 H3: 0.771, 0.969, 0.882 H4: 0.027, 0.299, 0.979 H5: 0.246, 0.031, 0.130

**Table 9:** Crystallographic data of calcium alanates.

Compound	Space Group, Cell Dimensions [Å] and Angles [°]	Atomic Coordinates
Ca(AlD_4_)_2_	*(Pbca)* No. 61 [221] a = 13.4491(27); b = 9.5334(19); c = 9.0203(20) α = β = γ=90	Ca: 0.8958(1), 0.4662(2), 0.2818(3) Al1: 0.4389(3), 0.7757(5), −0.0011(8) Al2: 0.8460(3), 0.1060(4), 0.1839(5) D1: 0.3710(9), 0.6842(11), 0.1087(12) D2: 0.5280(8), 0.8546(12), 0.0825(14) D3: 0.4877(9), 0.6706(12), −0.1183(13) D4: 0.3647(8), 0.8817(11), −0.0835(13) D5: 0.8264(10), 0.0829(11), 0.0086(8) D6: 0.8094(8), 0.2610(8), 0.2337(14) D7: 0.9590(5), 0.0702(12), 0.2407(16) D8: 0.7762(9), −0.0075(10), 0.2636(16)
CaAlD_5_	*(P 2_1_/c)* No. 14 [221] a = 9.8000(19); b = 6.9081(13); c = 12.4503(23) α = 90; β = 137.936(4); γ = 90	Ca1: 0.7845(16), 0.2166(19), 0.7382(13) Ca2: 0.3275(14), 0.2676(16), 0.1816(11) Al1: 0.8017(15), 0.3097(16), 0.4907(12) Al2: 0.2071(14), 0.2175(14), 0.8706(11) D1: 0.0058(17), 0.3009(19), 0.5190(14) D2: 0.6406(16), 0.4242(18), 0.3076(12) D3: 0.6070(14), 0.2725(17), 0.4696(13) D4: 0.7010(18), 0.3865(14), 0.8592(15) D5: 0.9589(14), 0.1915(15), 0.6767(10) D6: 0.1259(17), 0.0329(14), 0.9070(13) D7: 0.1154(19), 0.3773(14), 0.9139(15) D8: 0.2848(16), 0.0634(15), 0.8156(14) D9: 0.2612(19), 0.4064(13), 0.8154(13) D10: 0.4470(13), 0.1884(16), 0.0707(12)

**Table 10:** Crystallographic data of strontium-aluminum hydrides.

Compound	Space Group, Cell Dimensions [Å] and Angles [°]	Atomic Coordinates
Sr(AlH_4_)_2_	*Pmmn* (No. 59) [44] a = 9.1165(18); b = 5.2164(11); c = 4.3346(8) α = β = γ = 90	Sr: 0.1958(3), ¼, ¾ Al1: 0.9665(11), ¼, ¼ Al2: 0.37309(11), ¾, ¼
SrAlD_5_ (experimental)	*Pbcm* (No. 57) [229] a = 4.6226(10); b = 12.6213(30); c = 5.0321(10) α = β = γ = 90	Sr: 0.2532(7), 0.8925(3), ¼ Al: 0.3296(11), 0.1597(3), ¼ D1: 0.4366(13), ¼, 0 D2: 0.3461(13), 0.5790(5), ¼ D3: 0.0311(13), 0.7146(3), ¼ D4: 0.1914(7), 0.0718(3), 0.4986(9)
SrAlH_5_ (theoretical)	*P 2_1_2_1_2_1_* (No. 19) [192] a = 12.679; b = 5.200; c = 4.508 α = β = γ =90	Sr: 0.908, 0.104, 0.036 Al: 0.165, 0.117, 0.071 H1: 0.763, 0.859, 0.278 H2: 0.078, 0.337, 0.918 H3: 0.093, 0.860, 0.945 H4: 0.079, 0.114, 0.374 H5: 0.254, 0.116, 0.768
Sr_2_AlD_7_	*I2* (No. 5) [230] a = 12.552(1); b = 9.7826(8); c = 7.9816(7) α = γ = 90; β = 100.286(4)	Sr1: 0.0935(3), 0.3289(4), 0.3195(6) Sr1´: 0.9065(3), 0.6711(4), 0.3195(6) Sr2: 0.8609(4), 0.0684(4), 0.0882(6) Sr2´: 0.1391(4), 0.9316(4), 0.4118(6) Al1: 0.671(1), 0.847(1), 0.232(2) Al1´: 0.329(1), 0.153(1), 0.268(2) D1: 0.7494(7), 0.8594(7), 0.077(1) D1´: 0.2506(7), 0.1406(7), 0.423(1) D2: 0.6014(7), 0.7106(7), 0.117(1) D2´: 0.3986(7), 0.2894(7), 0.383(1) D3: 0.7658(6), 0.7378(8), 0.341(1) D3´: 0.2342(6), 0.2622(8), 0.159(1) D4: 0.5885(6), 0.8298(8), 0.379(1) D4´: 0.4115(6), 0.1702(8), 0.121(1) D5: 0.7395(6), 0.9919(7), 0.3291(9) D5´: 0.2605(6), 0.0081(7), 0.1709(9) D6: 0.5748(6), 0.9558(7), 0.1157(8) D6´: 0.4252(6), 0.0442(7), 0.3843(8) D7: 0.4375(6), 0.6037(7), 0.3189(9) D7´: 0.5625(6), 0.3963(7), 0.1811(9)
SrAl_2_D_2_	*P-3m1* (No. 164) [233] a = b = 4.5253(1); c = 4.7214(2) α = γ = 90; β = 120	Sr: 0,0,0 Al: 0.3333, 0.6667, 0.4589(7) D: 0.3333, 0.6667, 0.0976(4)

**Table 11:** Crystallographic data of barium-aluminum hydrides.

Compound	Space Group, Cell Dimensions [Å]	Atomic Coordinates
BaAlH_5_	Pna21 (No. 33) [207] a = 9.1568; b = 7.0718; c = 5.1039 α = β = γ = 90	Ba: 0.686, 0.156, 0.256 Al: 0.041, 0.846, 0.229 H1: 0.008, 0.946, 0.905 H2: 0.584, 0.844, 0.025 H3: 0.578, 0.786, 0.504 H4: 0.357, 0.695, 0.233 H5: 0.708; 0.545, 0.214
Ba_2_AlD_7_	I2/a (No. 15) [235] a = 13.197(3); b = 10.237(2); c = 8.509(2) α = γ = 90; β = 101.290(9)	Ba1: 0.3459, 0.5848, 0.3249 Ba2: 0.1084, 0.3247, 0.0852, Al1: 0.927, 0.096, 0.235 D1: 0.004(1), 0.116(1), 0.077(2) D2: 0.846(1), 0.974(1), 0.135(2) D3: 0.023(1), 0.999(2), 0.325(2) D4: 0.844(1), 0.104(2), 0.387(2) D5: 0.983(1), 0.249(2), 0.324(2) D6: 0.832(1), 0.207(1), 0.115(2) D7: 0.693(1), 0.864(1), 0.322(2)

**Table 12:** Lanthanides-Aluminum Hydrides (MAlH_6_) relevant data [261].

Material	Hydrogen Content	Hydrogen Release *	Decomposition Temperature [°C]	Crystal Structure (*R-3m*, No.166) [Å]	
	[wt.%]			Experimental	DFT **
LaAlH_6_	3.51	0.98	Beginning 100, ending 240	a = 6.4732 c = 6.2765	a = 6.5272(4) c = 6.3212(7) H: 0.2149, 0.7851, 0.4904
CeAlH_6_	3.49	0.80	First step: Beginning 100, ending 170 Second step: Beginning 180, ending 270	a = 6.4711 c = 6.2527	a = 6.4637(4) c = 6.2609(7) H: 0.2147, 0.7853, 0.4910
PrAlH_6_	3.47	0.78		a = 6.4217 c = 6.2028	a = 6.4106(7) c = 6.2118(11) H: 0.2139, 0.7861, 0.4894
NdAlH_6_	3.41	0.78		a = 6.3796 c = 6.1616	a = 6.3846(7) c = 6.1741(10) H: 0.2132, 0.7868, 0.4883

* (including NaCl load) ** M = La, Ce, Pd, Nd on 0, 0, 1/2, and Al on 0, 0, 0.

**Table 13:** Crystallographic data of Europium alanates [44].

Compound	Space Group, Cell Dimensions [Å]	Atomic Coordinates
Eu(AlH_4_)_2_	*Pmmn* (No. 59) a: 9.1003(13); b: 5.1912(8); c: 4.2741(5)	Eu: 0.1966(3), 0.25, 0.75 Al: 0.9625(12), 0.25, 0.25 Al: 0.3821(5), 0.75, 0.25
EuAlH_5_	*Pnma* (No. 62) a: 12.481(3); b: 5.0103(12); c: 4.5887(11)	Eu: 0.6517(3), 0.25, 0.2016(12) Al: 0.4105(14), 0.25, 0.586(4)

**Table 14:** Crystallographic data of Li-Na mixed alanates.

Compound	Space Group, Cell Dimensions [Å]	Atomic Coordinates
Na_2_LiAlD_6_ (experimental)	*Fm-3m* (No. 225) [284] a: 7.38484 (5)	Na: 0.25, 0.25, 0.25 Li: 0.5, 0.5, 0.5 Al: 0, 0, 0 D: 0.238(4), 0, 0
Na_2_LiAlH_6_ (calculated)	*P 2_1_/c* (No. 14) [282] a = 5.165; b = 5.251; c = 7.339 α = 90, β = 90.03, γ = 90	Li: 0, 0, 0.5 Na: 0.99, 0.47, 0.25 Al: 0, 0, 0 H: 0.07, 0.02, 0.23 H: 0.23, 0.3, 0.53 H: 0.2, 0.27, 0.96

**Table 15:** Crystallographic data of Li-K mixed alanates.

Compound	Space Group, Cell Dimensions [Å]	Atomic Coordinates
K_2_LiAlH_6_	*R-3m* (No. 166) [286] a: 5.62068(8) c: 27.3986(6)	Li: 0, 0, 0.4036(8) Al: 0, 0, 0 Al: 0, 0, ½ K: 0, 0, 0.1270(1) K: 0, 0, 0.2853 (1) H: 0.096(7), −0.096(7), 0.466(3) H: 0.205(5), −0.205(5), 0.638(2)
K_2_LiAlH_6_	*Fm-3m* (225) [267] a = 7.9383	K: ¼, ¼, ¼ Li: ½, ½, ½ Al: 0, 0, 0 H: 0.216, 0, 0
K_2_LiAlH_6_ (calculated)	*P 2_1_/n* (No. 14) [282] a = 5.528 b = 5.536 c = 7.832 α = 90, β = 90.03, γ = 90	K: 0, ½, ¼ Li: 0, 0, ½ Al: 0, 0, 0 H: 0, 0, 0.23 H: 0.27, 0.27, ½ H: 0.23, 0.23, 0

**Table 16:** Crystallographic data of Li-Mg mixed alanates.

Compound	Space Group, Cell Dimensions [Å]	Atomic Coordinates
LiMg(AlD_4_)_3_	*P 2_1_/c* (No. 14) [288] a = 8.37113(16) b = 8.73910(17) c = 14.3012(3) α = γ = 90, β = 124.8308(8)	Mg: 0.6305(6), 0.5292(4), 0.8833(3) Li: 0.127(3), 0.4720(19), 0.3822(14) Al1: 0.7615(5), 0.6282(4), 0.1512(3) Al2: 0.4745(5), 0.8809(4), 0.8581(3) A13: 0.9593(5), 0.2510(4), 0.4986(3) D1: 0.6057(14), 0.5722(12), 0.1782(9) D2: 0.6523(14), 0.5907(11), 0.0190(6) D3: 0.7843(17), 0.8088(9), 0.1721(10) D4: 0.9475(12), 0.5201(10), 0.2158(9) D5: 0.4888(15), 0.7127(10), 0.8153(9) D6: 0.6918(11), 0.9294(11), 0.9554(8) D7: 0.3783(15), 0.9895(12), 0.7474(8) D8: 0.3312(15), 0.8752(13), 0.8981(10) D9: 0.9500(15), 0.3124(13), 0.3908(8) D10: 0.7599(14), 0.1597(12), 0.4549(10) D11: 1.1293(13), 0.1222(10), 0.5635(8) D12: 0.9941(14), 0.3727(11), 0.5902(7)
LiMgAlD_6_	*P321* (No. 150) [290] a = b = 7.985550(2) c = 4.378942(7) α = β = 90, γ = 120	Mg: 1, 0.3570(13), 0 Li: 0, 0.686(6), ½ Al1: 0, 0, 0 Al2: 1/3, 2/3, 0.492(10) D1: 0.540(3), 0.763(2), 0.278(3) D2: 0.119(3), 0.576(2), 0.734(3) D3: 0.904(2), 0.117(2), 0.228(3)

**Table 17:** Crystallographic data of Li-Ca mixed alanates.

Compound	Space Group, Cell Dimensions [Å]	Atomic Coordinates
LiCa(AlH_4_)_3_ (experimental)	*P6_3_/m* (No. 176) [292] a = b = 8.91978(12); c = 5.8887(7) α = γ = 90, β = 120	Li: 0, 0, 0 Ca: 2/3, 1/3, ¼ Al: 0.2805(3), 0.9027(4), ¼
LiCa(AlH_4_)_3_ (theoretical)	*P6_3_/m* (No. 176) [293] a = b = 9.093 c = 5.996 α = γ = 90, β = 120	Li: 0, 0, 0 Ca: 2/3, 1/3, ¼ Al: 0.3, 0.9, ¼ H1: 0.544, 0.501, ¼ H2: 0.807, 0.815, ¼ H3: 0.535, 0.754, 0.029
LiCaAlH_6_ (theoretical)	*P–4* (No. 81) [294] a = b = 6.6652 c = 16.5607 α = γ = β= 90	Li1: 0, 0, 0 Li2: 0, 0, ½ Li3: ½, ½, 0 Li4: ½, ½, ½ Li5: 0, ½, 0.4843 Li6: 0, ½, 0.0085 Ca1: 0.3119, 0.2730, 0.1937 Ca2: 0.2380, 0.1803, 0.6978 Al1: 0.2812, 0.2452, 0.3777 Al2: 0.2812, 0.2398, 0.8264 H1: 0.4729, 0.2445, 0.3245 H2: 0.2947, 0.0386, 0.4346 H3: 0.1523, 0.0958, 0.2964 H4: 0.1623, 0.4222, 0.3053 H5: 0.2861, 0.4340, 0.4450 H6: 0.2449, 0.0032, 0.5840 H7: 0.0609, 0.3014, 0.8263 H8: 0.2532, 0.4729, 0.9283 H9: 0.3881, 0.3663, 0.7959 H10: 0.2891, 0.0307, 0.8161 H11: 0.2130, 0.1039, 0.9599 H12: 0.2158, 0.4740, 0.0859

**Table 18:** Crystallographic data of Na-K mixed alanates.

Compound	Space Group, Cell Dimensions [Å]	Atomic Coordinates
K_2_NaAlD_6_	*Fm-3m* (No. 225) [295] a = b = c = 8.118(1) α = β = γ = 90	K: ¼, ¼, ¼ Na: ½, ½, ½ Al: 0, 0, 0 D: 0.2167(8), 0, 0

**Table 19:** Representative infrared frequencies of Al-H bonds reported for different alanates.

Alanate	Mode/Peak Position [cm^−1^]			Comments/Reference
	Stretching	Bending	Librational	
LiAlH_4_	1779, 1642	885, 811, 715	465	Pure crystalline material [177]
	1800, 1780, 1645	890, 810, 700		[306] and Refs. within
	1757, 1615	900, 830		[310] and Refs. within
Li_3_AlH_6_	1410, 1300	1000, 960, 854		[321]
	1386, 1276	1000, 950, 850		[310] and Refs. within
Li_3_AlD_6_	1020, 915	740, 700, 635		[321]
NaAlH_4_	1680	900, 811, 730, 680		Pure crystalline material [177]
	1680	900, 800, 735, 690		[306] and Refs. within
Na_3_AlH_6_	1440, 1290	930, 842, 690		[321]
KAlH_4_	1715	811, 729		Pure crystalline material [177]
RbAlH_4_	1715	811, 763, 739		Pure crystalline material [177]
	1715	811, 769, 729		[306] and Refs. within
CsAlH_4_	1711	741		Pure crystalline material [177], Ref. [306] and Refs. within
Mg(AlH_4_)_2_	1935	800, 625		Ref. [306] and Refs. within
	642, 1937			[43]
	1620, 1700–1800			[39]
	2013, 1905, 1850, 716, 663, 620, 360, 302, 282			[210]
Ca(AlH_4_)_2_	600, 1780			[40]
	1788	816, 653	482	[306] and Refs. within

**Table 20:** Representative Raman frequencies of Al-H bonds reported for different alanates.

Alanate	Assignation/Peaks Position [cm^−1^]					Comments/Reference
	Combination	Stretching	Bending	Librational	Translational	
LiAlH_4_		1837, 1762, 1722	950, 882, 830, 780, 690	510, 438, 322	220, 165, 151, 143, 112, 95	[306]
Li_3_AlH_6_	2090, 1974	1604, 1311	1014, 975	577, 510		[321]
Li_3_AlD_6_	1478, 1397	1137, 940	730, 686	412, 360		[321]
NaAlH_4_		1762, 1681,	848, 817, 770	521, 429	180, 125, 117	[306]
Na_3_AlH_6_		1556, 1465, 1152, 1070	990, 815, 760	560, 480		[321]
KAlH_4_		1779, 1711	790			[306]
Mg(AlH_4_)_2_		1969, 1944, 1808	824, 768, 736			[306]
	2077, 1852, 1845, 812, 758, 742, 298, 232, 87					[310]

**Table 21:** Thermodynamic data of alanates.

Alanate	Formation Enthalpy $\Delta H_{f}^{0}\;[\mathrm{kJ}/\mathrm{mol}]$	Dehydrogenation Reaction/Dehydrogenation Enthalpy [kJ/mol]		Apparent Activation Energy [kJ/mol]
LiAlH_4_	−107.1 [330] −113.42 [81] ^‖^ −114.8 [87] ^‡^ −118.9 [331] −119 [71]	(15)	−10 [26] −9.79 [81] ^‖^	102 [75], 103 [332] (pure) 42.6 [73] (TiCl_3_-1/3AlCl_3_ 2 mol%) 67 [74] (NbF_3_ 1 mol%) 81.5 [333] (FeCl_2_ 2 mol%) 87.4 [308] (TiN 2 mol%)
Li_3_AlH_6_	−310.89 [81] ^‖^ −298.5 to −311.0 [81,87,330]	(16)	15.72 [81] ^‖^ 25 [26]	54.8 [73] (TiCl_3_-1/3AlCl_3_ 2 mol%) 77 [74] (NbF_3_ 1 mol%)
NaAlH_4_	−78.9 [334] −105.6 [268] −113.0 [331] −116.3 [335] ^‡^	(25)	36.7 [335] ^‡^ 37 [88] ѳ 36–40.9 [71] ֎ 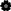	114.2 [336] (pure) 113.8 (NiFe_2_O_4_ 3 mol%) [315], and (MnFe_2_O_4_) [317] 86.4 [336] (LaCl_3_ 2 mol%)
Na_3_AlH_6_	−238.8 [335] ^‡^ −172.8 [334] ^‖^ −260 [337]	(26)	69.6 [335] ^‡^ 47 [88] ѳ 46.8–47 [71] ֎ 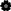	162.6 [336] (pure) 86.4 [336] (LaCl_3_ 2 mol%)
KAlH_4_	−166.6 [331] −183.7 [161] ֎ −128 [175] ^‖^	(34)	70 [167] 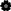 ~55 [168] ‖ 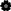	140 [164] (pure) 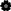 80 [164] (TiCl_3_ 2% mol) 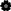
K_3_AlH_6_	−224.7 [175] ^‖^	(35)	81 [167] 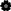	?
CsAlH_4_	−164.9 [330]	(39)	?	?
Mg(AlH_4_)_2_	−79 [338] (“assessed value”)	(42)	20.4 [43] (at 0 K, ab-initio)	123.8 [199] (pure not milled) 123.6 [195] (with LiCl_2_) 123 [197] (submicron rods) 82.3 [200], 85.5 [208] (TiF_4_ doped)
Ca(AlH_4_)_2_	−214 [192] ^‖^	(45)	−7 [224] 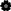 , −7.4 [220]	?
CaAlH_5_	−224 [192] ^‖^	(46)	26 [41] 32 [224] 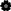 , 31.1 [220]	161 [41], 153.4 [219] (pure) 57.4 [219] (TiF_3_ 10 wt.%)
SrAlH_5_	−248 [192] ^‖^	(54)	?	?
BaAlH_5_	−224 [207] ^‖^	--	?	?
LaAlH_6_ [261]	?	?	~30 ‖, 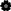	?
MAlH_6_, M=Ce, Pr, Nd [261]	?	(82)	~28-32 ‖, 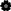	?
Eu(AlH_4_)_2_ [44]	?	?	−4.4 and 57 (for 2 consecutive reactions of hydrogen release, 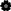 ).	?
Na_2_LiAlH_6_	−84.5 [268] 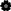 ^‖^ −55.26 [297] 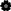 (^‖^, 300 K) −53.5 [267] 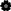	(88)	53.5 [267] 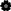 , 63.8 [274] 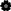 56.4 [276] 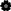 TiF_3_ doped 57.3 [285] TiF_4_ doped	173 [274] 143.6 [285] TiF_4_ doped
K_2_LiAlH_6_	−100.5 [268] 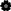 ^‖^ −102.42 [297] 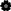 (^‖^, 300 K) −82 [267] 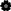	?	82 [267] 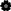	?
LiMg(AlH_4_)_3_	−192.6 [206] (^‖^, 0 K)	(91)	−4.16 [289] 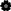 , −5 [339] 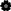	~66 277
LiMgAlH_6_	−184.8 [206] (^‖^, 0 K)	(92)	8.89 [289] 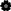 , 9 [339] 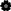	?
K_2_NaAlH_6_	−107.66 [297] 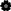 (^‖^, 300 K) −97 [267] 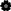	(96)	97 [267] 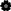 98 [295] TiF_3_ doped 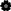	124.43 [296] 88.05 TiF_3_ catalyzed [296]

^‡^ CALPHAD ^‖^ DFT ^Ѳ^ Ti-doped ^֎^ (and references within) 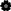 Explicitly reported per mole of H_2_, i.e., kJ/mol H_2_, ? Unknown.
